# Psychiatric readmissions and their association with physical comorbidity: a systematic literature review

**DOI:** 10.1186/s12888-016-1172-3

**Published:** 2017-01-03

**Authors:** Lilijana Šprah, Mojca Zvezdana Dernovšek, Kristian Wahlbeck, Peija Haaramo

**Affiliations:** 1 Research Centre of the Slovenian Academy of Sciences and Arts, Sociomedical Institute, Novi trg 2, 1000 Ljubljana, Slovenia; 2 National Institute for Health and Welfare, Mental Health Unit, P.O. Box 30, 00271 Helsinki, Finland

**Keywords:** Mental disorders, Readmission, Physical comorbidity, Multimorbidity, Systematic review

## Abstract

**Background:**

Comorbidity between mental and physical disorder conditions is the rule rather than the exception. It is estimated that 25% of adult population have mental health condition and 68% of them suffer from comorbid medical condition. Readmission rates in psychiatric patients are high and we still lack understanding potential predictors of recidivism. Physical comorbidity could be one of important risk factors for psychiatric readmission. The aim of the present study was to review the impact of physical comorbidity variables on readmission after discharge from psychiatric or general inpatient care among patients with co-occurring psychiatric and medical conditions.

**Methods:**

A comprehensive database search from January 1990 to June 2014 was performed in the following bibliographic databases: Ovid Medline, PsycINFO, ProQuest Health Management, OpenGrey and Google Scholar. An integrative research review was conducted on 23 observational studies.

**Results:**

Six studies documented physical comorbidity variables only at admission/discharge and 17 also at readmission. The main body of studies supported the hypothesis that patients with mental disorders are at increased risk of readmission if they had co-occurring medical condition. The impact of physical comorbidity variables on psychiatric readmission was most frequently studied in in patients with affective and substance use disorders (SUD). Most common physical comorbidity variables with higher probability for psychiatric readmission were associated with certain category of psychiatric diagnoses. Chronic lung conditions, hepatitis C virus infection, hypertension and number of medical diagnoses were associated with increased risk of readmission in SUD; Charlson Comorbidity Index, somatic complaints, physical health problems with serious mental illnesses (schizophrenia, schizoaffective disorder, personality disorders); not specified medical illness, somatic complaints, number of medical diagnoses, hyperthyroidism with affective disorders (depression, bipolar disorder). Co-occurring physical and mental disorders can worsen patient’s course of illness leading to hospital readmission also due to non-psychiatric reasons.

**Conclusions:**

The association between physical comorbidity and psychiatric readmission is still poorly understood phenomenon. Nevertheless, that physical comorbid conditions are more common among readmitted patients than single admission patients, their association with readmission can vary according to the nature of mental disorders, characteristics of study population, applied concept of comorbidity, and study protocol.

**Electronic supplementary material:**

The online version of this article (doi:10.1186/s12888-016-1172-3) contains supplementary material, which is available to authorized users.

## Background

Comorbidity conditions have been studied from the perspectives of different outcomes, one of them being readmission after hospital discharge [1–3] and could be an important risk factors associated with readmission for people with psychiatric disorders. However, this issue remains poorly understood.

It is estimated that almost one in seven persons hospitalised for psychiatric reasons are readmitted within 1 month of discharge [4]. Since readmission rates in psychiatric patients are high, it is of great interest to determine potential predictors of such recidivism. Psychiatric patients have been widely reported to be at an increased risk of morbidity and mortality due to physical disorders [5–7]. A serious and persistent mental disorder can result in patient’s losing up to four years of life, compared to individuals without mental disorder. Suicide, cancer, accidents, liver disease, and septicaemia increase premature mortality among persons with serious and persistent mental disorder [8].

The results of conducted research on comorbidity influenced as well the classification systems of mental disorders by pointing out, that current psychiatric diagnoses are not discrete entities and most patients with one diagnosis also fulfil the diagnostic criteria for another diagnosis, implying that comorbidity of related disorders is rather a rule than exception [9]. Heterogeneous category of diagnoses / diseases by using exclusion criteria show hierarchy between diagnoses, and related clinical entities lead to frequent co-occurrence of diagnoses of mental disorders [10].

In the 2001–2003 US National Comorbidity Survey Replication (NCS-R), a representative epidemiological survey revealed that comorbidity between medical and mental disorders is the rule rather than the exception [11, 12]. More than 68% of adults with a mental disorder (diagnosed with a structured clinical interview) reported having at least one general medical disorder, and 29% of those with a medical disorder had a comorbid mental health condition. Elderly patients and those with diagnoses of organic brain syndromes reportedly having the highest risk for comorbid medical illness [13]. Thus, there is an indication that having a mental disorder is a risk factor for physical disorder and vice versa. For example, having a physical illness is one of the strongest risk factors for depression; and depression is also a risk factor for physical illness [14, 15]. Among respondents in the 1999 epidemiological National Health Interview Survey (NHIS; an ongoing national household survey of non-military and noninstitutionalized persons in the United States) the likelihood of having major depression diagnosed (via a screening instrument) increased with each additional comorbid chronic medical condition [16]. In other studies, depression is reported to be comorbid with 26 disease categories and is most prevalent in combination with gastrointestinal diseases, stroke, musculoskeletal diseases, Parkinson’s disease, respiratory diseases, and obesity [17]. A study by Andres et al. [18] revealed that in addition to survival risks associated with post-myocardial depression in patients with recurrence of acute myocardial infarction (AMI), psychiatric disorders influenced the consecutive readmission for AMI with the same severity as did tobacco, diabetes, and obesity.

A growing body of evidence demonstrates that certain physical conditions are observed with increased frequency in patients with severe mental illness [1, 19–21]. As summarized by de Hert et al. [5], there is very good or good evidence for increased risk for various physical diseases in patients with mental disorders, for example, human immunodeficiency virus (HIV), impaired lung function, obstetric complications, stroke, myocardial infarction (MI), hypertension, obesity, diabetes mellitus to name a few.

Unfortunately, several authors reported that clinicians fail to recognize these comorbid medical illnesses in nearly half of all cases [22, 23]. In a number of patients, physical illness could then lead to psychiatric conditions themselves, or worsening of existing symptoms. As well as the mental disorder itself, adverse effects of medications or other treatments can result in serious medical pathology [24]. It seems that the physical health of people with a severe mental illness has been neglected for decades, and still is today [5, 6].

In the literature we can notice a diverse use of terminology for mental and physical health conditions: mental disorder, mental illness, mental impairment, psychiatric disorder, psychological disorder, somatic condition, medical condition, physical illnesses, etc. In our study, we mainly used terms: mental and physical disorders, unless when referring to studies where authors or the context required different terminology. Mental disorders comprise a broad range of problems, with different symptoms (reflecting in various categories of diagnoses/diseases). However, they are generally characterized by some combination of abnormal thoughts, emotions, behaviour and relationships with others. For the purposes of our literature review it was most suitable to use the term mental disorders allowing us to include different characteristics of psychiatric patients described in reviewed studies (e.g., diagnoses, symptoms, diseases, etc.).

### The concept of comorbidity

The term “comorbidity” is well-recognised in research and clinical settings, but the concept remains rather complex and methodological approaches differ. Approaches to study the impact of comorbidity become challenging also due to the lack of consensus about how to define and measure the concept of comorbidity [27].

The concept of comorbidity was established by Feinstein in 1970 [25] to denote cases in which a “distinct additional clinical entity” occurs during the clinical course of a patients’ index disease. Later on, more complex concepts of comorbidity were developed intended for use in clinical setting, research and health care management and planning [26]. There is currently no consensus around the definition of comorbidity, which can be defined in several different ways. Consequently, clinicians, researchers and managers are using different comorbidity concepts when faced with co-occurring chronic diseases, disorders, health conditions, illnesses or health problems. Overall, the term comorbidity has three meanings [19]: a) Indicating a medical condition in a patient existing simultaneously but independently with another condition; b) Indicating a medical condition in a patient that causes, is caused by, or is otherwise related to another condition in the same patient; c) Indicating two or more medical conditions in a patient that exist simultaneously, regardless of their causal relationship.

An increasing interest in the subject as well as methodological obstacles in analysing data on comorbidity has resulted in the first comprehensive trial of integrating different aspects of comorbidity definitions [27]. Authors combined different constructs and measures associated with the core concept of comorbidity, the coexistence of two or more conditions in a patient. In this respect, four major distinctions were made according to the nature of the health condition, the relative importance of the co-occurring conditions, and the chronology of the conditions: comorbidity, multimorbidity, morbidity burden and patient’s complexity.

The Charlson and Elixhauser comorbidity measures are of the most frequently used methods in the comparative research on comorbidity, reflecting the morbidity burden [28–32]. The Charlson Comorbidity Index predicts the ten-year mortality for a patient in relation to a range of comorbid conditions.

The Elixhauser comorbidity measure developed a list of 30 comorbidities relying on the ICD-9-CM coding manual. The comorbidities were not simplified as an index as each comorbid condition may affect several outcomes (length of hospital stay, hospital changes, and mortality) differently among diverse groups of patients [33]. Both, the Charlson and the Elixhauser indices were originally used to predict mortality for inpatient populations, but t have also been applied to outpatient populations to measure other health outcomes in the clinical research (prediction of service use, readmission risk, health costs, etc.) [31, 33–35].

Since each construct of comorbidity illuminates different aspects of morbidity it is important to distinguish between them, mostly because of their use in research, clinical practice, and management of services [27]. For instance in clinical research, the construct of choice will be determined by its ability to inform patient management. Although the perception of patient complexity is relevant to all aspects of care, the construct of comorbidity, with its emphasis on an index disease, may be predominantly useful in specialist care, whereas multimorbidity and morbidity burden may prove better constructs for primary care. From an epidemiological and public health perspective, the constructs of comorbidity and multimorbidity are of greatest interest, while morbidity burden and patient complexity seems to be more suitable from the health services research and policy perspective [27, 31].

### Outcome research and comorbidity

The comorbidity between mental and somatic disorders is an important field in everyday medical practice, and is becoming widely recognised also in psychiatry [5, 36]. There is growing interest among practitioners and researchers in the impact of comorbidity on a range of outcomes, such as mortality, health-related quality of life, patient's functioning, and health care utilization [37]. Readmission after psychiatric hospitalization is commonly used as a quality of care indicator by government funding agencies, policy-makers, and hospitals deciding on clinical priorities [38].

Comorbidity issues are also linked with higher economic burden since the increased direct health costs (usually represent the costs associated with medical resource utilization, including the consumption of inpatient, outpatient, and pharmaceutical services within the health care delivery system) and indirect health costs (defined as the expenses incurred from the cessation or reduction of work productivity as a result of the morbidity and mortality associated with a given disease, typically consist of work loss, worker replacement, and reduced productivity from illness and disease), are also associated with treatment of patients with more chronic condition [39]. For example, about 80% of Medicare spending is devoted to patients with four or more chronic conditions, with costs increasing exponentially as the number of chronic conditions increase [40, 41].

Since physical comorbidity could be an important risk factor for readmission, much effort has been put into developing reliable risk prediction models for hospital readmission whereas physical comorbidity have been integrated as well [42]. Authors emphasized that the majority of the 26 readmission risk prediction models, studied within the systematic review, have poor predictive ability [42]. Physical comorbidities, basic demographic data, and clinical variables have proved to much better predict mortality than readmission risk. Namely, hospital and health system-level factors, social, environmental, and medical factors (e.g., the timeliness of post-discharge follow-up, coordination of care with the primary care physician, the supply of hospital beds, access to care, social support, substance abuse, and functional status) can also contribute to readmission risk; however the utility of such factors has not been widely studied. Authors concluded that the inclusion of such factors could conceivably improve the predictive ability of prediction models for readmission risk [42]. Recently a new risk tool was introduced: READMIT - A clinical risk index to predict 30-day readmission after discharge from acute psychiatric units by Vigod et al. [43]. A comprehensive risk tool consists of several variables, independently associated with one month readmission: repeat admissions, emergent admissions, diagnoses, unplanned discharge, medical comorbidity (including Charlson Comorbidity Index), prior service use intensity and time in hospital. Their study confirmed the medical comorbidity as a significant risk factor in predicting of 30-day readmission [43].

In patients with comorbidities besides higher risk of dying, a poorer functional status or quality of life also a greater use of health services has been reported [44, 45]. These findings led to the conclusion that among patients with comorbidity, the focus of health care should not only be on one specific disease, but also on the pathology in other organs and on indicators for quality of care such as complications of treatment, readmissions, treatment strategies and compliance to generally accepted clinical guidelines. In order to improve outcomes and reduce medical costs, a better understanding of the associations between physical comorbidities and psychiatric readmissions is needed. Namely, from a clinical or a policy decision-making point it would be very useful to be able to identify those patients with high risk of readmission in order to ensure a better follow-up of mental and somatic disorders after discharge, or to be able to calculate standardized readmission rates as indicators of quality of health care.

This systematic review belongs to a series of reviews from the Comparative Effectiveness Research on Psychiatric Hospitalisation (CEPHOS-LINK) project on determinants of readmission after discharge from psychiatric hospital care. The main objective of this study was to review and describe the effect of physical comorbidity variables on readmission after discharge from psychiatric or general health inpatient care with a psychiatric diagnosis.

## Method

### Search methods for identification of studies

Comprehensive literature searches were conducted in the electronic bibliographic databases Ovid Medline, PsycINFO, ProQuest Health Management and OpenGrey. In addition, Google Scholar was utilized. Relevant publications published between January 1990 and June 2014 were included.

Studies on the association between mental health and readmission were searched using combinations of keywords (used as MeSH terms or free text, depending on the database) describing mental health services and readmission. For more detailed description of the search terms please see Additional files 1 and 2 (Detailed search strategies and Detailed search strategy for articles on physical comorbidity). In addition, the reference lists of all included articles were manually checked for additional studies.

### Criteria for considering studies for review on physical comorbidity

Studies on readmission (to a psychiatric or non-psychiatric bed) after discharge from psychiatric, general or specialised inpatient care were included in this review. The original discharge had to be one with a main psychiatric diagnosis and additional medical diagnoses (both diagnosed using for example the ICD-10 system [49]) or medical conditions relevant for physical comorbidity. Admissions to day hospitals or community programmes were not considered as readmissions.

Quantitative longitudinal studies were selected for this systematic review, including both observational and intervention studies. Qualitative studies, case reports, papers not including original data, such as editorials, letters to the Editor and commentaries were excluded. The same applies to the studies that were not published as full reports. Three review papers were retrieved from initial search. They were excluded because physical comorbidity was not included among reviewed characteristics of psychiatric readmission.

Several medical conditions relevant for physical comorbidity (physical comorbidity variables) were considered at admission, at discharge and at readmission. They can be grouped into three core categories:

a) Medical diagnoses (according to codes from International Classification of Diseases – ICD codes, DSM IV / Axis III (medical condition) classification) [47]

b) Physical conditions (specified medical illnesses without classification codes e.g., cardiovascular disease, cardiac problems, diabetes, trauma, nutritional and metabolic diseases, etc.)

c) Variables describing the burden of medical illness indicated as “Number of medical diagnoses”, “Physical health problems”, “Charlson Comorbidity Index”, “Number of somatic complaints”.

Only studies examining adult populations (age ≥ 18 years) were included in the review. In the case of studies examining also adolescents we included these studies in the review if the reported mean age in the cohort was at least 18 years.

A primary outcome of interest was related to the existence or not of a link between physical comorbidities and readmission to inpatient hospital care (psychiatric or non-psychiatric/general), and the studies that did not report results on readmission were thus excluded.

In addition we included in the review also studies that addressed physical comorbidity only at admission / discharge. This aspect of the review was carried out due to the fact that we were attentive also in identifying which variables of physical comorbidities were observed in association with psychiatric conditions in order to identify those possible specific physical conditions that may be related to certain mental disorder.

**Fig 1:**
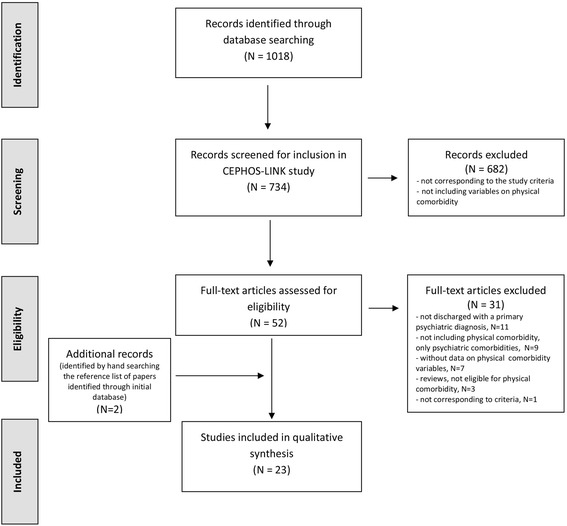
Flow of studies on psychiatric readmission and their association with physical comorbidity. Eligible studies on physical comorbidity were retrieved by the systematic literature search and article selection process, consisting of the following steps: identification of records, screening for inclusion, assessing of studies eligibility, and inclusion of studies into the integrative research review

No restrictions regarding language or publication status were used in the original searches. However, a few studies had to be excluded from the final examination because translation was not available into any of the language mastered by the multi-lingual research team (e.g., from Chinese). In the end, all but one of the included studies were written in English. The only non-English study was in Spanish. The flow of studies through the selection process is detailed in Fig. 1 the PRISMA flow-chart; [48].

### Data collection and analysis

Two pairs of researchers [LŠ, RS and VD, EL] independently screened all abstracts. Full-texts were screened, if necessary to establish the eligibility of articles. In a subsequent step full text of all candidate papers were retrieved and independently screened by two researchers [LŠ, MZD]. Discrepancies were resolved by discussion by these two researchers, until agreement on inclusion or exclusion of the study was reached.

Available structured data on physical comorbidity variables associated with readmission were extracted from the included studies and entered into an evidence evaluation table independently by two researchers [LŠ, MZD]. The evidence evaluation table included the following information: study period, study design, type of study (observational/ interventional), characteristics of study population, time to follow-up, inclusion/exclusion criteria, main outcomes, number of participants, age and gender distribution in the data, included diagnostic groups/diagnostic distribution, physical comorbidity variables included in the study, readmission rate, used readmission time/time since discharge, key factors affecting readmission, mortality rate, description of performed statistical analyses, and countries in which the included studies were carried out.

An integrative research review was conducted since meta-analysis was not feasible due to the heterogeneity of the studies and low number of data observations associated with physical comorbidity variables.

## Results

### The selection process of the included studies

Although, psychiatric readmission was studied in different clinical settings and diagnostic groups of mental disorders, several studies included the presence of physical comorbidities within exclusion criteria, considering them as cofounders. Of the 734 unique articles identified in the initial search only 52 were included in the review. After additional screening and selection, a further 31 full-text studies were excluded due to following reasons:

- not discharged with a primary psychiatric diagnosis, *n* = 11
- not including physical comorbidity, only psychiatric comorbidities (F diagnoses), *n* = 9
- without specified data on physical comorbidity variables, *n* = 7
- reviews, not eligible for physical comorbidity, *n* = 3
- not corresponding to the study criteria on physical comorbidity, *n* = 1

Through subsequently hand searching of the reference list in included papers, identified through initial database, two additional eligible articles on physical comorbidity have been retrieved. Finally, 23 full-text articles (all with observational type of studies) were included for full text assessment of eligibility and into integrative review (Fig. 1).

### Overall description of reviewed studies

Key characteristics of the studies selected for the systematic review on physical comorbidity are presented in the Additional file 3: Table S1, Additional file 4: Table S2 and Table 1.

**Table 1:** Summary table of general characteristics and results from studies included in a systematic literature review on psychiatric readmissions and their association with physical comorbidity

	N of studies	Comments
Publication date	1991 - 1999: 6 2000–2009: 11 2010–2013: 6	4 studies: 2011 2 studies/year: 2009, 2006, 2000,1997 1 study in all other years
Country	USA: 14 Canada: 2 Denmark: 2 Australia: 2 UK: 1 Spain: 1 Japan: 1	61% of included studies were carried out in USA
Source of data	Hospital medical records only: 10 Big administrative database (National registries): 7 Hospital medical records + Interviews and/or Self- assessment questionnaires, Clinical assessment instruments: 6	44% of included studies used data from hospital medical records
Applied statistical method /model	Only descriptive statistics / nonparametric test: 4 Bivariate / Multivariate statistical analysis: 19	83% of included studies applied Bivariate / Multivariate statistical analyses
Study population according to age	Age range: 18 – 80+ Studies focusing on elderly population: 5	22% of included studies focused on elderly population
Study population according to gender	Both: 21 Only female: 1 Only man: 1	In 2 studies with veterans were included males or females only, recruited from Veterans hospital
Categories of psychiatric diagnoses included in the study (as index disease)	Affective disorders: 7 Substances use disorders: 7 All psychiatric diagnoses: 6 Serious mental illness: 3	30,5% of studies recruited patients with affective and substances use disorders; 26% with all psychiatric diagnoses; 13% with serious mental illness Affective disorders include: depression, bipolar disorder Serious mental illness include: schizophrenia, schizoaffective disorder, bipolar disorder, personality disorders
Types of physical comorbidity variables included in studies	Specified physical comorbidity (with ICD codes): 6 Specified physical comorbidity (without ICD codes): 6 Charlson Comorbidity Index : 5 Not specified physical comorbidity: 5 Number of medical diagnoses and somatic complaints: 3	In 2 studies were applied two types of physical comorbidity variables within the same study protocol: Charlson Comorbidity Index + Specified physical comorbidity (with ICD codes) / Specified physical comorbidity (without ICD codes)
Most common physical comorbidity variables documented at admission/discharge in the category ”Affective disorders ”	*Medical diagnoses/illness:* Medical illness (not specified), Diabetes, Hyperthyroidism, Obesity, Cardiovascular disease, Hypertension, High cholesterol *Variables describing the burden of medical illness*: Somatic complaints (excessive concern with body or functional capacity), The number of medical diagnoses, Charlson Comorbidity Index	
Most common physical comorbidity variables documented at admission/discharge in the category “Substances use disorders ”	*Medical diagnoses/illness*: Chronic lung conditions, Asthma, Hepatitis C virus (HCV) infection, Hepatitis B,HIV(+), Epilepsy, Hypothyroidism, Hypertension, Skin and subcutaneous tissue diseases, Infectious parasitic diseases, Digestive diseases, Cardiac problems/angina, Cirrhosis, Gastritis, Diabetes, Pregnancy, Accidental poisonings, Adverse drug reactions, Accidental falls *Variables describing the burden of medical illness*: Physical health problems, Charlson Comorbidity Index, Number of medical diagnosis	
Most common physical comorbid variables documented at admission/discharge in the category “All psychiatric diagnoses ”	*Medical diagnoses/illness*: Cellulitis, Chronic obstructive pulmonary disease, Liver disease, Diabetes, Hypertension, Circulatory heart conditions, Epilepsy, Hypothyroidism *Variables describing the burden of medical illness*: Charlson Comorbidity Index, Number of medical diagnoses	
Most common physical comorbidity variables documented at admission/discharge in the category “Serious mental illness ”	*Variables describing the burden of medical illness:* Non specified physical health problems, Charlson Comorbidity Index	None of reviewed studies in the category “Serious mental illness“did document medical diagnoses/illness
Data on mortality	NO: 19 YES: 4	83% studies did not analyse mortality
Readmission time	12 months: 5 1 month: 3 6 months: 2 3 months: 2 24 months: 2 26 months: 2 48 months: 2	Other less frequently used time frames for readmission time: less than 1 month (8–30 days), 58 months, 70 months, 79 months, 84 months, 192 months, 216 months, 240 months
The impact of physical comorbidity variables on readmission	YES: 12 NO: 4 NA: 7	52% of included studies did show that physical comorbidity may be associated with hospital readmission 6 studies documented physical comorbidity only at admission/discharge without including them at readmission
Most common physical comorbidity variables with higher probability for readmission according to categories of psychiatric diagnoses	*Affective disorders*: Not specified medical illness, Somatic complaints, Number of medical diagnoses, Hyperthyroidism *Substances use disorders*: Chronic lung conditions, Hepatitis C virus (HCV) infection, Hypertension, Number of medical diagnoses *All psychiatric diagnoses*: Cellulitis, Chronic obstructive pulmonary disease, Liver disease, Diabetes, Hypertension, Circulatory heart conditions *Serious mental illness:* Higher Charlson Comorbidity Index, Somatic complaints, Physical health problems	
Applied concept of comorbidity construct	Multimorbidity: 11 Patient’s complexity: 7 Morbidity burden: 4 Comorbidity: 1	*Applied concept of comorbidity construct according to categories of psychiatric diagnoses*: Affective disorders: Morbidity burden N = 3; Multimorbidity N = 2; Patient’s complexity N = 1; Comorbidity N = 1 Substances use disorders: Multimorbidity N = 6; Patient’s complexity N = 1 All psychiatric diagnoses: Patient’s complexity N = 4; Multimorbidity N = 2 Serious mental illness: Multimorbidity N = 1; Patient’s complexity N = 1; Morbidity burden N = 1

^a^Comorbidity: presence of additional diseases in relation to an index disease in one individual

Multimorbidity: presence of multiple diseases in one individual

Morbidity burden: overall impact of the different diseases in an individual taking into account their severity

Patient’s complexity: overall impact of the different diseases in an individual taking into account their severity and other health-related attributes

In general, included studies (*n* = 23) documented physical comorbidity variables at time of hospitalisation (admission, discharge, readmission). But physical comorbidity was not analysed in all studies from the perspective of psychiatric readmission. 17 studies reported on physical comorbidity at readmission (Additional file 3: Table S1, Additional file 4: Table S2; studies listed from No 1. to 17.). In view of this, we included in the review also those studies (*n* = 6) that addressed physical comorbidity (regardless the type of the physical comorbidity variable – diagnoses, numbers the physical disorders, Charlson Comorbidity Index, etc.) only at admission / discharge. Above studies didn’t report the potential associations between physical comorbidities and psychiatric readmission, since physical comorbidities were only recorded at the time of the initial admission with a descriptive objective (Tables Additional file 3: Table S1, Additional file 4: Table S2; studies listed from No 18. to 23.). Besides reviewing studies according to physical comorbidity issue, they were further analysed from the perspective of constructs covering different aspects of comorbidity (described in the Introduction chapter) [27]. Namely, comorbidity, multimorbidity, morbidity burden, patient’s complexity, implies a different understanding of the concept of comorbidity (Additional file 3: Table S1 and Table 1).

### General characteristics of the reviewed studies

Out of 23 reviewed studies 17 were published after year 2000, the oldest published in the year 1991 and most recent, published in the year 2013 (Table 1). The largest number (n = 4) of included studies originated from year 2011. According to the geographical scope of conducted studies, 61% of reviewed studies were carried out in USA, two in Canada, Denmark, Australia, and in United Kingdom, Spain, and Japan (Additional file 3: Table S1 and Table 1).

The majority of included studies (44%) obtained data from hospital medical records only, 31% from big administrative database (national registries) and 26% of studies combined data from hospital medical records and interviews and/or self- assessment questionnaires and clinical assessment instruments (Table 1). According to the applied statistical method, 83% of included studies used bivariate / multivariate statistical analyses (Additional file 3: Table S1).

In one third of reviewed researches (*n* = 7) a study population consisted of patients with affective disorders (predominantly with depression, followed by bipolar disorder). Another seven studies included patients with substance use disorders (SUD), six studies included all psychiatric diagnoses and three studies focused on patients diagnosed with serious mental illnesses (SMI; schizophrenia, schizoaffective disorder, bipolar disorder, personality disorders).

Most of studies (92%) included both genders. One study was restricted to female s only [69], and one study only included a male study population [68]. The study restricted to female population included a group of female veterans discharged from Veterans Affairs Hospital and the male study involved male veterans of either World War II or the Korean War, treated at the Houston Veterans Affairs Medical Center. Age of study populations in reviewed publications ranged from 18 – 80+. Five studies were focused only on elderly population [50, 55, 59, 63, 68] (Table 1).

The periods of follow-up varied from less than 1 month (*n* = 1) to more than seven years (*n* = 4). Most frequently reported follow-up periods were 12 months (*n* = 5) and one month (*n* = 3). More than 80% of reviewed studies (*n* = 19) did not document mortality rates during the follow- up period. Among studies that monitored mortality, rates depended considerably on the length of follow-up period, age range of the study population and burden of comorbid psychiatric and physical conditions [52, 55, 57, 59].

Physical comorbidity variables, identified in the 23 reviewed studies, are summarized in Additional file 3: Table S1 and Table 1. Variables were classified according to the physical conditions relevant for physical comorbidity and reflect medical illnesses (medical diagnoses according to ICD codes and listed medical problems without codes) and the burden of medical illness (indicated as number of medical diagnoses, somatic complaints, Charlson Comorbidity Index) co-occurring with psychiatric condition. Six studies documented physical comorbidity variables only at admission /discharge, and 17 studies as well at readmission.

A supplementary evaluation of applied constructs covering different aspects of comorbidity was conducted in order to ascertain which aspects of comorbidity have been addressed. Evaluation revealed that all studies did not follow to the same concept of comorbidity. The majority of studies (48%) were based on multimorbidity concept (presence of multiple diseases in one individual). Patient’s complexity (overall impact of the different diseases in an individual taking into account their severity and other health-related attributes) was the next most frequent applied concept (31% of studies). Morbidity burden concept (overall impact of the different diseases in an individual taking into account their severity) was applied in 17% of studies. The least frequently used concept was comorbidity (presence of additional diseases in relation to an index disease in one individual), applied only in one study (in 4% of all included studies). Concepts of comorbidity construct differed also according to categories of psychiatric diagnoses. Morbidity burden prevailed in category of affective disorders, whereas multimorbidity construct in SUD. More detailed description of applied comorbidity constructs can be seen in Additional file 3: Table S1 and Table 1.

### Physical comorbidity variables in patients with mental disorders

An analysis of co-occurring physical and mental disorders was carried out in order to identify those physical variables that most commonly co-occur with certain mental disorders, as well to identify which of specified physical comorbidity variables might have a potential impact on hospital readmission (Additional file 3: Table S1, Additional file 4: Table S2 and Table 1).

Comorbidity physical variables were widely documented in a form of classification codes (6 studies) and specified medical illnesses without classification codes (6 studies), followed by Charlson Comorbidity Index (5 studies), not specified health problems (5 studies) and number of medical diagnoses/somatic complains (3 studies). Overall, several studies reported that patients with mental disorders had more physical comorbidities compared to those without mental disorders conditions [52, 63, 65, 69] (Additional file 3: Table S1 and Table 1).

Multimorbidity concept was used in almost half of studies and frequently applied in retrospective cohort studies based on medical records from large administrative databases or national patient registries [2, 34, 35, 57, 59, 65]. Patient’s complexity concept was applied in one third of reviewed studies, acknowledging that morbidity burden is influenced not only by health-related characteristics, but also by socioeconomic, cultural, environmental, and patient behaviour features. For instance, the study of Mark et al. [2] revealed that social factors have been found to contribute to 39% of admissions in patients with SMI, followed by factors related to mental and physical disorders (31%) and dangerousness to self or others (20%). Aggressive behaviour, self-injurious behaviour and sexually inappropriate behaviour co-occurring with physical health deterioration in patients with learning disabilities have been reported as risk factors for hospital readmission [58]. Also the following patient related factors were found as significant predictors of readmission : residential instability, alcohol as a primary drug of choice, single marital status, unemployment, multiple drug use, an older age, ethnicity, treatment incompletion, care distress, maladaptive family functioning, poorer psychosocial functioning [50, 54, 55, 67].

Several physical disorders have been described in admitted patients with main psychiatric diagnosis (Additional file 4: Table S2 and Table 1). The following most common physical conditions (medical diagnoses/illness) were found in some categories of mental disorders at hospital admission / discharge:

1. All psychiatric diagnoses: cellulitis, chronic obstructive pulmonary disease, liver disease, diabetes, hypertension, circulatory heart conditions, epilepsy, hypothyroidism [2, 51, 65, 68];
2. Affective disorders: diabetes, hyperthyroidism, obesity, cardiovascular disease, hypertension, high cholesterol [56, 57, 66];
3. Substances use disorders: chronic lung conditions, asthma, hepatitis C virus (HCV) infection, hepatitis B, HIV(+), epilepsy, hypothyroidism, hypertension, skin and subcutaneous tissue diseases, infectious parasitic diseases, digestive diseases, cardiac problems/angina, cirrhosis, gastritis, diabetes, pregnancy, accidental poisonings, adverse drug reactions, accidental falls [52, 53, 59, 61, 67, 69].

Physical comorbidity variables associated with burden of medical illness were documented in all categories of mental disorders in form of: Charlson Comorbidity Index, number of medical diagnoses, physical health problems and somatic complaints (Additional file 3: Table S1 and Table 1).

### The influence of physical comorbidity on readmission of patients with mental disorders

Out of 17 studies which documented physical comorbidity variables at readmission, 12 demonstrated that physical comorbidity may be associated with hospital readmission while four studies did not show that medical comorbidity is linked with a higher risk for readmission [51, 54, 55, 57]. Summarised results on the effects of most frequent reported physical comorbidity variables on readmission in patients with main psychiatric diagnosis are presented in Table 1. More detailed report on results from the reviewed studies is presented in Additional file 4: Table S2. Below are the key findings:

Physical disorders were more common among readmitted patients than single admission patients, nevertheless their impact on readmission varied according to the nature of mental disorders, characteristics of study population and study protocol (e.g., the duration of follow up period, index population, inclusion/exclusion criteria, etc.). In general, the main body of study outcomes support the hypothesis that patients with mental disorders were at increased risk of readmission if they had co-occurring medical conditions [3, 33, 61, 63]. Mercer et al. [68] reported that psychiatric patients were found to have approximately four times more psychiatric hospitalizations than medical hospitalizations despite the existence of multiple physical disorders in this population. Physical health problems contributed to the decision to readmit (readmission time: 36 months) in 16.5% of admissions of patients with SMI [62].

Physical comorbidity was not associated with psychiatric readmission in two studies [54, 55]. The negative associations between physical comorbidities and the probability of psychiatric readmission were identified in two studies, revealing that comorbidity with medical condition did reduce the readmission risk by 41% of psychiatric patients [51], and that less medical diagnoses increased the risk of mental disorder readmissions [59].

In almost all categories of psychiatric diagnoses (Affective disorders, SUD, SMI) the following physical comorbidity variables indicated a higher probability for readmission: no specified medical illness, more physical health problems, more somatic complaints, more medical diagnoses and higher Charlson Comorbidity Index score [35, 51, 62].

Several medical diagnosis/ physical disorders were reported to be associated with hospital readmissions in patients with main psychiatric diagnosis (Additional file 4: Table S2 and Table 1). Some of the physical comorbid conditions were found to increase the probability of readmission, like chronic lung conditions and hepatitis C virus infection in patients with SUD diagnosis [52, 60] and hypertension in patients with mental and/or SUD [2]. The study from Mai et al. [65] stated that patients with mental health disorders were about twice as likely as non-mental health patients to experience potentially preventable hospitalisations that accounted for more than 10% of all hospital admissions/discharges in this study population. Diabetes and its complications, adverse drug events, COPD, convulsions and epilepsy, and congestive heart failure have been the most common causes. For almost all comorbid conditions evaluated in the study of Mark et al. [2], a larger percentage of patients who were readmitted with mental and/or SUD diagnosis (readmission time: 8–30 days) had a comorbid condition compared with those who were not readmitted. The largest percentage difference has been reported for cellulitis, COPD, liver disease, diabetes, hypertension, and circulatory heart conditions.

Some studies indicated that the presence of mental disorder could worsen patient’s physical health or course of illness, consequently leading to hospital readmission due to non-psychiatric reasons. Thomsen & Kessing [56] reported that patients with bipolar disorder were found at greater risk of subsequent hospitalization (readmission time: 58 months, 70 months, 79 months) with hyperthyroidism in comparison with patients with depressive disorder. Also age was shown as important factor associated with poorer patient’s physical health. Kessing et al. [57] revealed that patients in age groups between 45 and 80 years of age discharged with a diagnosis of mania/bipolar disorder had a slightly increased rate (not significant) of getting a diagnosis of diabetes at readmission (readmission time: 240 months) whereas younger and older patients with mania/bipolar illness had a slightly decreased rate of diabetes.

## Discussion

This systematic review was conducted in order to synthesize the available research data on medical and physical comorbidity as risk factors that could be linked with hospital readmission of patients with comorbid psychiatric and medical conditions. Accordingly the relationships between psychiatric diagnoses and specific physical comorbidities that have been identified through this review only refer to hospitalized patients. Our literature review, irrespective of very diverse applied approaches in reviewed studies and limited generalizability, revealed also some recognizable trends in mental and physical disorder conditions.

Among 734 records identified through database searching only 23 studies documented physical comorbidity as a variable which was analysed at admission/discharge of patients with the main psychiatric diagnosis. Of these, 17 studies documented physical comorbidity also at readmission. Thus, several studies on psychiatric readmission included data on physical comorbidity within exclusion criteria. Some studies did check the Charlson Comorbidity Index at admission/discharge, predominantly to ensure that studied groups of patients did not significantly differ in medical comorbidity as authors considered it as a confounding variable [64–66]. Since our interest was also to examine if there are any specific physical conditions that may be related to particular mental disorders, we included 6 studies in our review where medical problems were recorded only at admission / discharge without being analysed from the perspective of readmission risk. In 23 of the reviewed studies we found a variety of applied aspects regarding comorbidity construct, selection of index population, source of data, outcome measures and research questions, study design, duration of follow up period, patient’s sociodemographic characteristics, etc. The majority of papers were not representative of the general psychiatric population discharged from an inpatient service. Generalizability is limited since reported results from several papers can be considered as biased according to: a) included categories of psychiatric diagnoses (only particular diagnoses were included from the whole psychiatric admitted population); b) gender inclusion (some studies were performed with only or predominantly in male or female groups of patients; c) age range (some studies included only a specific age-group e.g., the elderly); d) inclusion of different follow-up periods after discharge (from less than one month to several years); e) association of readmission risk with implemented study design (e.g., different inclusion / exclusion criteria, applied statistical models and source of data); f) scarce data on medical condition of included populations; g) geographical scope of included studies (uneven inclusion of studies from different countries, e.g., 61% of included studies in the review were performed in USA); h) applied concept of comorbidity (different models have been used with different types of variables, e.g., number of medical diagnoses, Charlson Comorbidity Index, specified medical diagnoses with or without ICD codes, etc.).

### Complex pathways of comorbid mental and physical disorder conditions

Studies included in this systematic review reported a broad spectre of co-occurring physical and mental disorder ‘conditions. Physical conditions consisted mainly of chronic noncommunicable disorders: cardiovascular disease, hypertension, diabetes, hyperthyroidism, hypothyroidism, high cholesterol, obesity, cellulitis, chronic lung conditions, chronic obstructive pulmonary disease, asthma, hepatitis C virus (HCV) infection, hepatitis B, HIV(+), epilepsy, skin and subcutaneous tissue diseases, infectious parasitic diseases, digestive diseases, liver disease, gastritis. The examined mental disorder conditions fell predominantly into the category of chronic, disabling and prevalent mental disorders: SUD, mood disorders (major depression, bipolar mood disorder), SMI (schizophrenia, bipolar mood disorder, schizoaffective disorder and personality disorders).

The pathways leading to comorbidity of mental and physical disorders are in several aspects interrelated. A broader insight into the dynamic of mental and physical comorbid conditions and its consequences can be reached when also taking into account outcomes from studies which examined readmission risk in patients with medical index disease and comorbid mental disorder. Two main characteristics can be noticed in the literature in this respect:

Firstly, the pathways leading to comorbidity of mental and physical disorders are complex and often bidirectional [70]. Epidemiological studies have been important in examining these pathways. For instance, physical conditions with a high symptom burden, such as migraine or back pain, might lead to depression [71] while major depression could represent a risk factor for developing a physical condition, such as cardiovascular disease [72].

Secondly, the course of comorbid mental disorder and physical conditions could be influenced by each other, leading to a worsening of either mental disorder and/or physical condition, consequently leading to hospital readmission due to non-psychiatric reasons. That could be demonstrated through proxy: longer hospital stay, frequent hospital readmission and increased mortality. For example, persons with bipolar mood disorder had a more severe course of disease, a higher total number of in-hospital deaths and a substantial higher burden of comorbidities [73]. Wells et al. [74] reported that depressive symptoms had an independent additive effect on the physical and social functioning of patients with chronic medical illness. Bipolar disorder was found at greater risk of subsequent hospitalization with hyperthyroidism [55]. Increased hospital mortality and readmission risk in patients with comorbid heart condition and depression were described in some other studies [18, 46, 75].

### The influence of comorbidity physical variables on readmission

Patients with mental disorders have been recognized in several studies as a vulnerable population for increased risk of readmission if they had co-occurring medical conditions [33, 35, 50, 60, 61, 63]. However, some studies in our review did not show that trend. In the study of Jaramillo et al. [51] it was demonstrated that having comorbidity with any medical condition reduces the readmission risk. Authors associated the protective effect of the medical comorbidity presence with two possible causes: a) most patients had comorbid epilepsy or thyroid problems, conditions which, if not properly controlled increase the risk of decompensation of psychotic or affective; b) having a medical condition may be related to better adherence to treatment, taking into account the possibility that the patient does not have the stigma of psychiatric diagnosis. In the study of Brennan et al. [59] a similar trend was observed, indicating that the burden of medical disease not necessarily increases the psychiatric readmission, since less medical diagnoses did increase the risk of mental disorder readmissions in elderly with SUD diagnosis in both genders.

Co-occurring psychiatric and physical conditions are described as a common condition also in studies with medical inpatients as index populations [36]. A range of studies revealed that pre-existing or co-occurring mental disorder may worsen the course of medical illness and can be seen as risk factor for readmission. For example in the recent study of Ahmedani et al. [76], the rate of readmission in patients with heart failure, acute MI, and pneumonia was 5% greater for individuals with a psychiatric comorbidity. Some studies reported that the risk of rehospitalisation among patients with COPD was increased in subjects with anxiety [77] and that patients hospitalized with a primary medical diagnosis and any co-occurring SMI were more likely to experience a subsequent medical hospitalization [78].

Regardless of the 52% of studies included in a systematic literature review showing that physical comorbidity may be associated with hospital readmission, it should be noted that the most common physical comorbidity variables with higher probability for readmission were mostly associated with specific categories of psychiatric diagnoses (Table 1). Thus non specified medical illness, somatic complaints, number of medical diagnoses and hyperthyroidism were associated with higher readmission risk in patients with main psychiatric diagnosis of depression or bipolar disorder. Discharged patients with SMI diagnoses and a higher Charlson Comorbidity Index score, somatic complaints and physical health problems have been reported at increased risk of subsequent hospital admission. Chronic lung conditions, hepatitis C virus (HCV) infection, hypertension and number of medical diagnoses were associated with readmission risk in patents with SUD.

### Methodological issues in studies with comorbid conditions

The comorbidity between mental and physical disorders is an important field in everyday medical practice and it is recognised as important topic in psychiatry. Notably in psychiatric practise the term comorbidity can also be used to indicate the coexistence of two or more psychiatric diagnoses which is arguably inappropriate. Because in most cases it is unclear whether the coexisting diagnoses actually reflect the presence of distinct clinical entities or refer to multiple manifestations of a single clinical entity. In psychiatric classification, comorbidity does not necessarily indicate the presence of multiple diseases, but instead can reflect current inability of psychiatrists to supply a single diagnosis that accounts for all symptoms [79].

Studies included in the present review addressed co-occurring psychiatric and physical conditions within a constructs which are related to different aspects of comorbidity [27]: comorbidity, multimorbidity, morbidity burden and patient’s complexity, implying a diverse understanding of comorbidity variables that might affect the readmission. This fact requires some caution in generalizing and understanding of the nature of the co-occurring mental and physical disorder conditions and their potential impact on hospital readmissions. The review revealed that different constructs of comorbidity were applied which limits a comparison of results on the possible impact of physical comorbidity regarding the psychiatric readmissions. In addition, authors did not describe why they selected particular comorbidity construct. Possibly that also the availability of data source influenced their choice.

Studies on comorbidity may be hampered by the so-called Berksons bias [80]. Patients who have been diagnosed with a disorder (e.g., depression) have greater chances of being diagnosed with a second disorder (e.g., diabetes) compared to subjects for whom no diagnosis has been made, as a doctor sees patients more often. Only one study [56] applied this criterion in the research protocol where patients with osteoarthritis were chosen as a control group due to its chronic and progressive nature, and because the disease and the treatment do not, as far as known, cause any biological affection on the brain and mood.

### Study limitations

Prospective studies on readmission in patients with co-occurring physical and mental disorders are not rare, but only a few examined the association between physical conditions and psychiatric readmissions. In the reviewed studies outcomes varied considerably, possibly because of differences in applied methods, data collection, definition of comorbidity and the number of chronic conditions included in analysis. In this regard, more high quality research is needed in the future to understand the associations between physical comorbidities and psychiatric readmissions.

Two main limitations of the present literature review need to be acknowledged. Firstly, although the methods for searching the literature were valid, we cannot be certain that all relevant studies on co-occurring psychiatric and medical conditions associated with readmission have been identified. Secondly, in the review included studies addressing co-occurring psychiatric and physical conditions within different comorbidity constructs. This circumstance requires some caution in terms of generalizing of results since small number of studies has been retrieved (*n* = 23), with diverse study protocols, different concept of comorbidity, index population, and follow up periods.

Since, to our knowledge, there are no previous systematic reviews in this area, this is the first systematic attempt taking into account all literature addressing the impact physical comorbidities on hospital readmission of patients with psychiatric diagnoses. The presented review covers publications over a more than 20 year period and provides a broad and systemised reporting of different aspects of co-occurring psychiatric and medical conditions in association with hospital readmission of patients with psychiatric diagnosis. In addition, the present systematic review addresses also different concepts on comorbidity. This provides an additional explanation on diversity of research results we are facing with, when co-existing physical and mental disorder conditions are studied in respect to hospital readmissions.

## Conclusions

Co-occurrence of mental and physical disorder conditions is very common in a clinical setting. However, the exact nature of the relationship between them is very complex and so far still not well understood. This vagueness is also reflected in the understanding of the influence that some physical comorbidities may have on psychiatric readmission. In this respect it is important to apply an adequate model of comorbidity, since various factors such as unhealthy lifestyle habits, psychotropic medication, and inadequate medical treatment or provision may have an important influence on readmission rates in psychiatric study population.

So far, very little work has been done on physical comorbid conditions among readmitted patients with mental disorders since comorbidity was seldom the main objective of studies, making it difficult to draw a solid conclusion about actual impact of physical comorbidity on readmission in psychiatric populations. Nevertheless, physical comorbid conditions seem to be more common among readmitted psychiatric patients than single admission patients, their association with readmission can vary according to the nature of mental disorders, characteristics of study population and study protocol.

The main body of reviewed studies supported the hypothesis that patients with mental disorders are at increased risk of readmission if they had a co-occurring medical condition, higher Charlson Comorbidity Index score, in and more medical diagnoses. Additionally, comorbidity is generally associated with mortality, quality of life, and health care but the consequences of specific disease combinations depend on many issues. The scarcity of eligible studies on psychiatric readmission and its association with physical conditions became apparent during performance of this review. It may be related also to the fact that several studies in this field did include the presence of physical comorbidities within the exclusion criteria. Namely, at admission/discharge have been documented several different types of physical comorbidity variables mainly in order to describe the study population, or to ensure that included samples matched in main medical conditions, or to describe a basic medical characteristics of index population. Due to the importance of the physical comorbidity issue in patients with mental disorders it would be advisable to include more variables on physical comorbidity in the future outcome research of mental disorders in naturalistic setting.

The impact of physical comorbidity on psychiatric readmission is still insufficiently investigated problem. But there is a growing interest among practitioners and researchers in the impact of physical comorbidity on a variety of outcomes in mental disorders, such as mortality, health-related quality of life and health care spending, which is substantially higher for patients with comorbid conditions [39]. The comorbidity of mental and physical disorders is on the increase and as pointed out by Sartorius [81] this issue is becoming a main challenge to medicine in the 21st Century.

Future research should address these topics with more in-depth studies since new insights in this field could lead to better prevention strategies to reduce psychiatric readmissions. From a clinical perspective it would be very useful to be able to recognise high risks for readmission in order to ensure a better monitoring and treating psychiatric patients with co-occurring physical disorders.

## Additional files

Additional file 1: Detailed search strategies. Are represented detailed search strategies in term of combinations of keywords (used as MeSH terms or free text) applied in different databases. (DOCX 21 kb)
Additional file 2: Detailed search strategy for articles on physical comorbidity. Are listed physical comorbidity variables with corresponding keywords which were used for identification of studies addressing the topic of physical comorbidity in patients discharged from psychiatric or general health in-patient care with a psychiatric diagnosis. (DOCX 18 kb)
Additional file 3: Table S1. General characteristics of the reviewed studies included in a systematic literature review on psychiatric readmissions and their association with physical comorbidity. (DOC 86 kb)
Additional file 4: Table S2. Description of results from studies included in a systematic literature review on psychiatric readmissions and their association with physical comorbidity. (DOC 107 kb)

## Abbreviations

BDI: Beck Depression Inventory
CEPHOS-LINK: Project: “Comparative Effectiveness Research on Psychiatric Hospitalisation”
COPD: Chronic obstructive pulmonary disease
ECT: Electroconvulsive therapy
F: Female
HCV: Hepatitis C virus
HIV: Human immunodeficiency virus
ICD: International Classification of Diseases
M: Male
M: Male
MDD: Major depressive disorder
MHCs: Mental health clients
MI: Myocardial infarction
N: Numerus
NA: Not applicable
SMI: Serious mental illness
SUD: Substance use disorders
VA: Veterans Affairs
