# Supplementary material for: Psychiatric readmissions and their association with physical comorbidity: a systematic literature review
Source: BMC Psychiatry. 2017 Jan 3;17:2. doi: 10.1186/s12888-016-1172-3 (PMC5210297; doi:10.1186/s12888-016-1172-3)
Supplement: Additional file 1: — Detailed search strategies. Are represented detailed search strategies in term of combinations of keywords (used as MeSH terms or free text) applied in different databases. (DOCX 21 kb) [file 12888_2016_1172_MOESM1_ESM.docx]

**Additional file 1.** Detailed search strategies.

1. **Ovid Medline**

Search date June 4 2014

PSYCHIATRIC DISORDERS

1. exp "mental disorders" (MeSH) or "mentally ill" or *stress, psychological/

2. ("mental disorder*" or "mental illness" or "mentally ill" or "mental disease" or "psychological problem*" or psychiatr* or "mental health problem*").ti.

3. 1 or 2

AND READMISSION

4. "patient readmission" (MeSH)

5. exp "continuity of care" (MeSH)

6 ("rehospitali*" or "readmission" or "revolving door").ab

7. 4 or 5 or 6

8. 3 and 7

**Search results:**

| **#** | **Searches** | **Results** |
| --- | --- | --- |
| 1 | ("mental disorder*" or "mental illness" or "mentally ill" or "mental disease" or psychiatr*).ti. | 96314 |
| 2 | exp *"mental disorders"/ or exp *"mentally ill persons"/ | 783358 |
| 3 | 1 or 2 | 825084 |
| 4 | ("rehospitali*" or "readmission" or "repeated admission" or "revolving door").ti. | 2031 |
| 5 | *Patient Readmission/ | 3193 |
| 6 | 4 or 5 | 3756 |
| 7 | 3 and 6 | 743 |
| 8 | limit 7 to yr="1990 -Current" | 525 |
| 9 | (editorial* or letter* or news* or comment*).pt. | 1505334 |
| 10 | 8 not 9 | 490 |

1. **PsycINFO**

Search date June 6 2014

PSYCHIATRIC DISORDER

TI (mental* or "mental dis*" or "problem behav*" or "behav* problem*" or psychiatr* or psychological) OR KW (mental* or "mental dis*" or "problem behav*" or "behav* problem*" or psychiatr*)

AND READMISSION

TI(rehospitali* or readmission or "continuity of care" or "revolving door") OR KW(rehospitali* or readmission or "continuity of care" or "revolving door")

**Search results:**

S4 **Limiters** - Publication Year: 1990-2014 **Search modes** - Boolean/Phrase 241

S3 S1 OR S2 423

S2 ( (MM "Mental Disorders" OR MM "Adjustment Disorders" OR MM "Affective Disorders" OR MM "Alexithymia" OR MM "Anxiety Disorders" OR MM "Autism" OR MM "Chronic Mental Illness" OR MM "Dementia" OR MM "Dissociative Disorders" OR MM "Eating Disorders" OR MM "Elective Mutism" OR MM "Factitious Disorders" OR MM "Gender Identity Disorder" OR MM "Hysteria" OR MM "Impulse Control Disorders" OR MM "Koro" OR MM "Mental Disorders due to General Medical Conditions" OR MM "Neurosis" OR MM "Paraphilias" OR MM "Personality Disorders" OR MM "Pervasive Developmental Disorders" OR MM "Pseudodementia" OR MM "Psychosis" OR MM "Schizoaffective Disorder") OR (MM "Psychiatric Patients") ) OR ( TI ("mental disorder*" or "mental* ill*" or "psychiatric disorder*" or "psychiatric patient*") ) 185,653

S1 (TI(rehospitali* or readmission or "repated admission" or "repeated hospitali*" or "revolving door") OR SU("Psychiatric Hospital Readmission")) 1,263

1. **ProQuest Health Management**

Search date June 6 2014

PSYCHIATRIC DISORDERS

AB("mental health" or "mental dis*" or "problem behav*" or "behav* problem*" or psychiatric or psychological)

AND READMISSION

AB("rehospitali*" or "readmission" or "continuity of care" or "revolving door")

**Search results:**

S4 S3 limited to 1990-2014 37 references

S3 S1 AND S2 38 references

S2 (MJMESH.EXACT.EXPLODE("Patient Readmission:E.02.760.400.620") OR MJMESH.EXACT.EXPLODE("Patient Readmission:N.02.421.585.400.620")) OR ti((rehospitali* or readmission or "repated admission" or "repeated hospitali*" or "revolving door")) 882

S1 SU.EXACT("Mental disorders") OR (MJMESH.EXACT.EXPLODE("Mental Disorders Diagnosed in Childhood") OR MJMESH.EXACT.EXPLODE("Mental Disorders")) OR ti(("mental disorder*" or "mental* ill*" or "psychiatric disorder*" or "psychiatric patient*"))

1. **OpenGrey (formely SIGLE)**

Search date June 10 2014

PSYCHIATRIC DISORDERS

psychiatry OR "psychiatric patient" OR "mental disorder" OR "mental disease" OR "behavioral disorder"

AND READMISSION

rehospitalisation OR readmission OR "continuity of care" OR "revolving door"

**Search results:**

(psychiatr* OR mental*) AND (rehospitali* OR readmission OR readmitted OR “repeated admission” OR "revolving door") 3 references

1. **Google Scholar**

Search date June 4 2014

**Search results:**

limit to years 1990-

allintitle: rehospitalization psychiatric 49 references

allintitle: rehospitalization mental 21 references

allintitle: readmission mental 34 references

allintitle:readmission psychiatric 113 references

allintitle: "revolving door" mental 26 references

allintitle: "revolving door" psychiatric 14 references

allintitle: repeated admission mental 0 references

allintitle: repeated admission psychiatric 0 references
