# Supplementary material for: Psychiatric readmissions and their association with physical comorbidity: a systematic literature review
Source: BMC Psychiatry. 2017 Jan 3;17:2. doi: 10.1186/s12888-016-1172-3 (PMC5210297; doi:10.1186/s12888-016-1172-3)
Supplement: Additional file 2: — Detailed search strategy for articles on physical comorbidity. Are listed physical comorbidity variables with corresponding keywords which were used for identification of studies addressing the topic of physical comorbidity in patients discharged from psychiatric or general health in-patient care with a psychiatric diagnosis. (DOCX 18 kb) [file 12888_2016_1172_MOESM2_ESM.docx]

**Additional file 2.** Detailed search strategy for articles on physical comorbidity.

Articles dealing with the significance of physical comorbidity variables for continuity of care and readmission after discharge from psychiatric or general health in-patient care with a psychiatric diagnosis were extracted if addressing one or more of the following topics (keywords):

- Comorbidity (keywords: medical comorbidity, physical problems, physical illness, medical complications, somatic disease, multimorbidity, morbidity)
- Health status (keywords: chronic diseases, weight gain, weight, smoking, cigarette use, alcohol use, illicit substance abuse)
- Mortality (keywords: mortality rates, suicide)
- Consequence of medical treatment (keywords: medication side effects, iatrogenic)
- Trauma (keywords: poisoning, accidents, fall, fracture, injury)
- Nutritional and metabolic diseases (keywords: obesity, diabetes mellitus, diet, hyperlipidaemia, cholesterol, polyuria, polydipsia, vitamin deficiencies)
- Cardiovascular diseases (keywords: myocardial infarction, circulatory diseases, stroke, heart failure, blood pressure, heart problems, atherosclerosis, hypertension)
- Respiratory diseases (keywords: chronic obstructive pulmonary disease, chronic lung disease, pneumonia, asthma)
- Infections (keywords: tuberculosis, urinary infection)
- Cancer (keywords: cancer incidence, cancer, terminal illness)
- Endocrine diseases (keywords: endocrinologic, thyroid dysfunction, hyperprolactinemia)
- Neurologic diseases (keywords: tardive dyskinesia, Parkinson’s disease, increased pain tolerance)
- Musculoskeletal disorders (keywords: osteoarthritis, arthritis, fractures, back problems, pain)
- Gastrointestinal disorders (keywords: gastrointestinal episodes, gastroenteritis, irritable bowel syndrome)
- Other medical conditions relevant for physical comorbidity: pressure ulcer, liver disease, chronic renal impairment, renal failure, dialysis, HIV, hepatitis, increased infection risk
