# Supplementary material for: Psychiatric readmissions and their association with physical comorbidity: a systematic literature review
Source: BMC Psychiatry. 2017 Jan 3;17:2. doi: 10.1186/s12888-016-1172-3 (PMC5210297; doi:10.1186/s12888-016-1172-3)
Supplement: Additional file 3: Table S1. — General characteristics of the reviewed studies included in a systematic literature review on psychiatric readmissions and their association with physical comorbidity. (DOC 86 kb) [file 12888_2016_1172_MOESM3_ESM.doc]

**Table 1. General characteristics of the reviewed studies included in a systematic literature review on psychiatric readmissions and their association with physical comorbidity**

| **No** | **Reference** | **Country** | **Study population /** **Number of participants** | **Age (years)**  **[(M, SD); (age range)]** | **Gender (Male/Female;**  **N, %)** | **Categories of psychiatric diagnoses included in the study (as index disease)** | **Types of physical comorbidity variables included in the study** | **Readmission time after discharge** | **Applied statistical method / model** | **Applied concept of comorbidity** |
| --- | --- | --- | --- | --- | --- | --- | --- | --- | --- | --- |
| 1. | Mark et al, 2013  [3] | USA | Medicaid inpatients with a principal M/SUD diagnosis, discharged from the hospital in the study period  *N: 121 271* | 18-65+ | Both  (M: 45.4%) | All psychiatric diagnoses | Specified physical comorbidity (without ICD codes) | 8–30 days | Hierarchical logistical regression model | Multimorbidity |
| 2. | Kim et al, 2011  [35] | USA | Patients with depression discharged from psychiatric hospitalization  *N:*  *17 856 (case group)*  *35 511 (control group)* | Age at index discharge:  case group 50.5 (10.5)  control group 50.5 (10.2) | Both  (% gender NA, mostly man) | Affective disorders (depression) | Charlson Comorbidity Index | 3 months | Conditional logistic regression model | Multimorbidity |
| 3. | Ono et al, 2011  [50] | Japan | Patients discharged from a hospital; hospitalized for dementia (Alzheimer’s, vascular or other types of dementia)  *N:326* | Total: 81.3 (7.2)  M: 80.7(7.3)  F: 81.7 (7.2) | Both  (M: 40%) | Serious mental illness (dementia) | Not specified physical comorbidity | 3 and 4 -24 months | Binomial logistic regression with a forward stepwise method | Morbidity burden |
| 4. | Jaramillo et al, 2011  [51] | Spain | Patients discharged from the Mental Health Center (Clínica Nuestra Señora de la Paz) during the study period with all psychiatric diagnoses  *N: 218* | 40.3 (12.6) | Both  (F: 56.9%) | All psychiatric diagnoses | Specified physical comorbidity (with ICD codes) | 12 months | Prospective cohort study  Multivariate analysis Negative binomial regression model | Patient's complexity |
| 5. | Irmiter et al, 2009  [52] | USA | Psychiatric inpatients initially hospitalized in the study period and were subsequently rehospitalized (group1: those who had a SUD or a co-occurring disorder that was diagnosed at the baseline admission; group2: those who were diagnosed with a SUD at a subsequent hospitalization (after baseline); group3: those who never received a substance abuse diagnosis at any subsequent hospitalization)  *N: 1375 *Divided in three cohorts (1) those diagnosed with SUDs, including co-occurring disorders, during their first psychiatric hospitalization (250); (2) those diagnosed with a SUD at a subsequent hospitalization (170); and 3) those never diagnosed with a SUD (995)* | Group 1: 35 (12) Group 2: 37 (16) Group 3: 42 (19) | Both  Group 1(M: 54%), Group 2 (M: 48%), Group 3 (M: 38%) | Substances use disorders | Specified physical comorbidity (with ICD codes) | 192 months | Multivariate logistical regressions | Multimorbidity |
| 6. | Li et al, 2008  [53] | Canada | Clients with substance use disorders admitted to an inpatient medical withdrawal management program, Vancouver Detox (VD) were included in the study.  *Clients were classified into three groups:  1) No request group—if clients did not seek readmission to VD during the 1-year follow-up  2) Short-term readmission request group—if clients requested readmission to VD within 1 month after being discharged from the index admission  3) Long-term readmission request group—if clients requested readmission to VD between 2 and 12 months after being discharged from the index admission  *N: 1102* | 19 - 86 (median: 41) | Both  (M: 61%) | Substances use disorders | Specified physical comorbidity (with ICD codes) | 1 and 2-12 months | A multinomial logistic regression model | Multimorbidity |
| 7. | Irmiter et al, 2007  [34] | USA | Patients with serious mental illness (SMI- schizophrenia, schizoaffective disorder and bipolar disorder) discharged from inpatient psychiatric care settings in the Veterans Affairs (VA) health system in fiscal year.  *N:*  *8.187; 27% Reinstitutionalization to Other VHA institutions* | Reinstitutionalization to psychiatric inpatient: 47 (10)  Reinstitutionalization to Other VHA institutions: 52 (12) | Both  (M: 49%) | Serious mental illness (schizophrenia, schizoaffective disorder, bipolar disorder) | Charlson Comorbidity Index | 84 months | Multivariable Cox proportional hazards regressions | Multimorbidity |
| 8. | Clements et al, 2006  [54] | USA | Patients discharged from psychiatric hospital (primary diagnosis - Axis I DSM-IV)  *N:1034* | 18-30 (30.4%)  31-50 (56.8%)  51+ (12.9%) | Both  (M: 44.5%) | All psychiatric diagnoses | Not specified physical comorbidity | 12 months | Retrospective cohort analysis  A multiple informant approach | Patient's complexity |
| 9. | Morrow-Howell et al, 2006  [55] | USA | Patients with depression (all subtypes) discharged from geropsychiatric unit to a community setting  *N:199* | 76.2 (7.124) | Both  (M: 30.2%) | Affective disorders (depression) | Not specified physical comorbidity | 6 months | Univariate statistics  Multinomial logistic regression | Patient's complexity |
| 10. | Thomsen & Kessing, 2005  [56] | Denmark | Patients with first hospital admissions with the resulting index discharge main diagnoses of depressive disorder, bipolar disorder and osteoarthritis and readmission to hospital with a resulting discharge diagnosis of hyperthyroidism during the observational period  *N: 134 069 Patients with depression: 22 024  Patients with bipolar disorder: 3132*  *Patients with osteoarthritis: 108 913* | Median age at first discharge (25 and 75% quartiles) Patients with depression: 55.7 (41.0–69.4) Patients with bipolar disorder: 45.5  (31.0–59.6) Patients with osteoarthritis: 67.7 (56.8–75.6) | Both Patients with depression-  F: 66% Patients with bipolar disorder- F: 52% Patients with osteoarthritis -  F: 58% | Affective disorders  (depression, bipolar disorder) | Specified physical comorbidity (with ICD codes) | 58 months  70 months  79 months | Historical cohort study  Cox regression models | Comorbidity |
| 11. | Kessing et al, 2004  [57] | Denmark | Patients who have been hospitalised at least once for depressive or bipolar disorders as main diagnosis and readmitted with a diagnosis of diabetes. Inpatients with main diagnosis of osteoarthritis (readmitted with diabetes) were chosen as a control group  *N:*  *Depression: 29 035 Bipolar illness: 6706  Osteoarthritis: 108 525* | Median, quartiles: Depression: 56.6 (43.3–68.8)  Bipolar illness: 46.6 (33.9–59.6)  Osteoarthritis: 68.5 (57.7–76.1) | Both Depression -  F : 68.1% Bipolar illness -  F: 57.8% Osteoarthritis - F: 59.7% | Affective disorders (depression, bipolar disorder) | Specified physical comorbidity (with ICD codes) | 240 months | Nationwide case register study  Poisson regression model | Multimorbidity |
| 12. | Seager et al, 2000  [46] | UK | People with learning disabilities, discharged from two learning disability hospitals to social and residential care homes. The specific criteria used for this study from DC-LD include verbally aggressive behaviour, physical aggressive behaviour, self-injurious behaviour and sexually inappropriate behaviour  *N: 54* | 24 - 83 (M: 50) | Both  (M: 67%) | All psychiatric diagnoses | Specified physical comorbidity (without ICD codes) | 216 months | Descriptive statistics (frequencies) | Patient's complexity |
| 13. | Brennan et al, 2000  [59] | USA | Elderly (65+) Medicare inpatients discharged with substance abuse, dependence, psychosis diagnosis. * A comparison group: patients discharged alive from hospital in the study period that had no substance use disorder diagnoses were matched case-to-case on age, gender and race with elderly patients who had substance use disorders  *N:*  *M: 14.383; F: 8.385* | All: 72,9 M: 72.3  F: 73.9 | Both  (M: 63%) | Substances use disorders | Number of medical diagnoses and somatic complaints | 48 months | Logistic regression | Multimorbidity |
| 14. | Labbate & Doyle, 1997  [60] | USA | Patients admitted with MDD to a psychiatric ward in large military medical center during the study period. Two groups of patients: 1- cases (consecutive multiple admissions) and 2- comparison group of patients without readmission  *N:*  **593; Recidivists (46) Single admission (50); *Recidivists comprised 8% of all evaluable patients, and 21% patients had a history of more than one MDD admission.* | Recidivist: 45 (18)  Single admission: 37 (13) | Both  Recidivist- M: 50%; Single admission- M: 54% | Affective disorders (depression) | Not specified physical comorbidity | 48 months | A one-way analysis of variance and χ2 | Morbidity burden |
| 15. | Phibbs et al, 1997  [61] | USA | Included were participants with discharges from inpatient substance abuse treatment programs  *N: 313 886* | 42.66 (2.46) | Both  (% gender NA) | Substances use disorders | Specified physical comorbidity (with ICD codes) | 6 months | Logistic regression (case mix model) | Multimorbidity |
| 16. | Kent & Yellowlees, 1994  [62] | Australia | Patients with diagnoses of schizophrenia, schizoaffective disorder, bipolar disorder, personality disorders, considered as heavy users of mental health services. *Heavy users were defined as follows: 1) had psychiatric illness; 2) had been admitted three or more times to Gledsade hospital, over to three year study period; 3) had been identified by staff as requiring high level of input; 4) had used the inpatient or outpatient services provided by South Australian Mental Health Services for at least 75% of the study protocol  *N: 50* | All: 34.1  M: 34.1  F: 33.2 | Both  (M: 52%) | Serious mental illness (schizophrenia, schizoaffective disorder, bipolar disorder, personality disorders) | Not specified physical comorbidity | 36 months | Descriptive statistics (relative frequency) | Patient's complexity |
| 17. | Colenda et al, 1991  [63] | USA | Geriatric depressed patients and young adult depressed patients, who meet DSMIII-R criteria for unipolar major depression, with consecutive admissions to the Medical College of Virginia (MCV)  *N:*  *111 (56 LOGD patients, 17 EOGD patients, and 38 YAD patients)* | Age at index admission - LOGD: 72.3 (7); EOGD: 63.8 (8.6); YAD: 29.2 (5.5)  Age at first psychiatric readmission - LOGD: 70.8 (6.6): EOGD: 51 (6.7); YAD: 26.7 (6.5) | Both  LOGD (M: 16%) EOGD (M: 18%)  YAD (M: 34%) | Affective disorders (depression) | Number of medical diagnoses and somatic complaints | 24 months | Historical cohort study Nonparametric tests (Wilcoxon and the Kruskal-Wallis test, χ2) | Morbidity burden |
| 18. | Walley et al, 2012  [64] | USA | General medical inpatients, with a substance use diagnosis recorded upon discharge (17%).  *N: 738 Substance Use: 615*  *Substance Use - had at least one substance use discharge diagnosis: 123* | 47.8 (11.4) | Both  (M: 65%) | Substances use disorders | Charlson Comorbidity Index | 1 month | Observational cohort study  Poisson and binomial regression models | Multimorbidity |
| 19. | Mai et al, 2011  [65] | Australia | Mental health clients (MHCs) and non-MHCs discharged from hospitals in study period, diagnosed with PPH medical condition  *N:*  *MHCs: 139 208*  *non-MHCs: 294 180* | MHCs: 43.7 (18.6)  non-MHCs: 45.1 (19.7) | Both  MHCs- M: 40.3%  non-MHCs: M: 40.4% | All psychiatric diagnoses | Charlson Comorbidity Index  Specified physical comorbidity (without ICD codes) | NA | Population-based retrospective cohort study  Unadjusted (univariate analysis) and adjusted (multivariate analysis) | Multimorbidity |
| 20. | Hassan & Lage, 2009  [65] | USA | Patients with bipolar disorder who had been hospitalized for the treatment of bipolar disorder and given an outpatient antipsychotic prescription within two weeks of hospital discharge. All patients included in the study had at least 6 months of continuous enrolment in the same health care plan before and 12 months after the index prescription date.  *N: 1973* | 18–64 (39.5 ± 13.2) | Both  (M: 63.9% ) | Affective disorders (depression, bipolar disorder) | Charlson Comorbidity Index  Specified physical comorbidity (without ICD codes) | 12 months | A retrospective cohort design  A multivariate, stepwise logistic regression | Morbidity burden |
| 21. | Callaghan & Cunningham, 2002  [67] | Canada | Patients with the medical records of 2595 consecutive admissions to an inpatient mixed-gender, hospital based alcohol and drug detoxification unit (Prince George Detoxification / Assessment Unit) in the study period  *N: 1454* | M: 39.6 (11.4)  F: 35.2 (10.9) | Both  (M: 67%) | Substances use disorders | Specified physical comorbidity (without ICD codes) | 36 months | Multiple regression analyses | Patient's complexity |
| 22. | Mercer et al, 1999  [68] | USA | Geropsychiatric inpatients, with all diagnoses (male veterans of either World War II or the Korean War), treated at the Houston Veterans Affairs Medical Center (HVAMC) during the study period. Those, hospitalized during the 1.5-year study period comprised the study group and were matched on demographic variables including race, age, marital status, and education with patients who were not hospitalized during the study period  *N: 150*  *rehospitalized: 75*  *not hospitalized: 75* | 59-88 (70.9, 5.5) | Only male | All psychiatric diagnoses | Number of medical diagnoses and somatic complaints | 18 months | Logistic regression of modified and dichotomized clinical variables | Patient's complexity |
| 23. | Walker et al, 1995  [69] | USA | Female veterans discharged from Veterans Affairs hospital with diagnosis of substance abuse or dependence or substance-induced psychosis and the control group with those discharged female veterans who did not receive a substance related diagnosis  *N:*  *With diagnosis of substance abuse or dependence or substance-induced psychosis (1698) Without a substance related diagnosis (12 037)* | With diagnosis of substance abuse or dependence or substance-induced psychosis: 39.9 (12.6)  Without a substance related diagnosis: 53.9 (18.2) | Only female | Substances use disorders | Specified physical comorbidity (without ICD codes) | 12 months | Descriptive statistic (frequencies, %) | Multimorbidity |

*Comorbidity: presence of additional diseases in relation to an index disease in one individual.

Multimorbidity: presence of multiple diseases in one individual.

Morbidity burden: overall impact of the different diseases in an individual taking into account their severity.

Patient’s complexity: overall impact of the different diseases in an individual taking into account their severity and other health-related attributes.
