# Supplementary material for: Psychiatric readmissions and their association with physical comorbidity: a systematic literature review
Source: BMC Psychiatry. 2017 Jan 3;17:2. doi: 10.1186/s12888-016-1172-3 (PMC5210297; doi:10.1186/s12888-016-1172-3)
Supplement: Additional file 4: Table S2. — Description of results from studies included in a systematic literature review on psychiatric readmissions and their association with physical comorbidity. (DOC 107 kb) [file 12888_2016_1172_MOESM4_ESM.doc]

**Table 2. Description of results from studies included in a systematic literature review on psychiatric readmissions and their association with physical comorbidity**

| **No** | **Reference** | **The main interest of the study** | **Inclusion of physical comorbidity variables at readmission** | **Psychiatric diagnoses (variables)** | **Physical comorbidity variables** | **Study period / Duration of follow-up (years, months)** | **Readmission rate (%)** | **Physical comorbidity variables that had some effect on the readmission** | **OTHER RELEVANT STUDY OUTCOMES** |
| --- | --- | --- | --- | --- | --- | --- | --- | --- | --- |
| 1. | Mark et al, 2013  [3] | To examine hospital- and patient-level predictors of readmission rates for mental and/or substance use disorders (M/SUDs) | Yes | Principal M/SUD diagnosis: ICD-9-CM codes (295.xx through 314.xx inclusive)  Medicaid admissions with a principal M/SUD diagnosis that received particular types of procedures: psychological evaluation and testing (ICD-9-CM 94.0); somatotherapy (ICD-9-CM 94.2); individual psychotherapy (ICD-9-CM 94.3); other psychotherapy and counselling (ICD-9-CM 94.4); and alcohol or drug rehabilitation and/or detoxification (ICD-9-CM 94.6) *The admitting psychiatric conditions were examined using the AHRQ mental health clinical classification system and included: adjustment disorders; anxiety disorders; attention-deficit disorders; delirium, dementia, and amnesiac disorders; developmental disorders; behavioural disorders usually diagnosed in infancy, childhood, or adolescence; impulse control disorders not elsewhere classified; mood disorders; personality disorders; schizophrenia and other psychotic disorders; alcohol-related disorders; substance-use-related disorders; suicide and intentional self-inflicted injury; screening and history of mental and substance use disorders; and miscellaneous mental disorders | Included comorbidities: Cellulitis  Endocarditis  Cirrhosis  Pancreatitis  Chronic obstructive pulmonary disease  Cancer  Liver disease  Diabetes  Renal disease  Hypertension  Ischemic heart disease  Circulatory heart conditions | 1. February 2004 - 1. November 2009  Follow up: 1 month  Discharge period was divided into two parts: (1) days 0–7 were examined for evidence of patients receiving follow-up care (thus, readmissions during this time period were excluded) and (2) readmission in days 8–30 following discharge, which was the primary outcome | At hospital level: Among 171 hospitals, the median readmission rate (30-day readmission rates) was 11% (mean 11.3%, SD 7.5%). The bottom 20th percentile had a readmission rate less than 7%, and the top 90th percentile had a readmission rate of 18%. The range was 0 to 60% | For almost all comorbid conditions evaluated in the 180-day pre-period, a larger percentage of patients who were readmitted in days 8–30 had a comorbid condition compared with those who were not readmitted. The largest differences were for patients with a pre-hospitalization period diagnosis of: cellulitis, chronic obstructive pulmonary disease, substance use disorder (largest percentage difference, 38% vs 28%), liver disease, diabetes, hypertension, or circulatory heart conditions (all p 0.001)  Predictor of readmission for mental and/or substance use disorder (M/SUD), 8–30 days following discharge: having hypertension diagnosis on the index admission | *The patient level factors most highly associated with a readmission were a prior admission for M/SUD, meaning that being admitted in the 6 months prior to the index admission increases the probability of being readmitted by 5.7%.  *The next largest effects were for an index admission diagnosis of schizophrenia or another psychosis and medication fill for a substance use disorder in the pre-period. Having a M/SUD outpatient visit or visit to a CMHC in the 6 months prior to an index admission is associated with about a 1% increase in the probability of being readmitted |
| 2. | Kim et al, 2011  [35] | The study assessed whether increased frequency of clinical monitoring during the high-risk period of 12 weeks after discharge from a psychiatric hospitalization reduced subsequent rehospitalisation | Yes | ICD-9-CM diagnosis – patients with major depression; comorbidity diagnoses: substance use disorder, PTSD, personality disorder, anxiety disorder, bipolar disorder type II, suicide attempts (based on ICD-9-CM codes E950 – E959 and on ICD-10 codes X60 – X84) | Number of Charlson medical comorbidities | 1. April 1999 – 30. September 2004  Follow-up: 12 weeks (84 days) after discharge from index hospitalization | 2% of the initial cohort (N = 63 925; patients with depression) had at least two psychiatric rehospitalisation; they were included in the study as a case group | In patients with comorbid substance use disorder diagnosis three or more Charlson comorbid conditions increase a relative risk of psychiatric rehospitalisation (p<.002) | *More Charlson medical comorbidities in group of hospitalised veterans with depression  *Sign. lower mean rate of visits for monitoring during the high risk period after discharge for case group (rehospitalisation) than for control group (no rehospitalisation)  *For patients without a comorbid substance use disorder higher monitoring was associated with rehospitalisation, but for those with a comorbid substance use disorder, higher monitoring was not associated with rehospitalisation  *Persons of middle age (35-64) were less likely than younger persons (<35) to be hospitalized |
| 3. | Ono et al, 2011  [50] | The aim of study was to investigate the factors related to readmission to a ward for dementia patients with special attention to sex-related issues | Yes | Dementia psychiatric diagnoses (DSM-IV-TR) | Physical comorbidity variable: yes/no | 1. April 2000 – 31. March 2008  Follow-up: 24 months after discharge | Total readmission: 23.3%; M: 25%, F: 22.3% | Most of the women showing early readmission had comorbidity  High physical function was related to late readmission in women | *Small number of cohabitants and outcome were factors related to early readmission in men; outcome and long stay in the ward were related to early readmission in women  *Care distress and short stay in the ward were related to late readmission in women |
| 4. | Jaramillo et al, 2011  [51] | Evaluating the effect of demographic, clinical and treatment factors on rehospitalisation | Yes | All psychiatric diagnoses, divided into:  A) Axis I: bipolar disorder, major depression, schizophrenia-like disorders, medical comorbidity, anxiety disorder, substance use, other;  B) Axis II: Group A, Group B, Group C, organic disorder, lack of diagnosis (dominant diagnostic characteristics: bipolar disorder -29.8% and no Axis I diagnosis - 67.7%) | Medical comorbidity within DSM IV / Axis III classification (25.7% had some type of medical comorbidity classifiable in the DSM IV axis III, mainly epilepsy, hypothyroidism and hypertension) | 1. August -  18. December 2009  Follow up: 12 months | 57.8% of patients were readmitted at least once during the year following the index hospitalization | To have comorbidity with any medical condition reduces of readmission risk by 41%. | *The variable most strongly associated with the rehospitalisation is the diagnosis on Axis I substance use (this diagnosis increases the expected number of readmissions to a 122.5%)  *Demographic variables affecting number of readmissions in either analysis: age, gender, socioeconomic status |
| 5. | Irmiter et al, 2009  [52] | To examine predictors for patients with substance use disorders (SUD) at baseline hospitalization and subsequent rehospitalisation | Yes | Psychiatric ICD 9 codes: (1) Schizophrenia Disorders (schizophrenia and schizoaffective diagnoses),  (2) Mood Disorders (depression and bipolar diagnoses),  (3) Other Psychiatric Disorders (anxiety, eating, psychosis, etc.), and (4) Substance Use Disorders (all substance intoxication, all substance abuse and dependence, etc.) | Medical ICD 9 codes:  (1) Endocrine, Nutritional, Metabolic, and Immunity Disorders,  (2) Circulatory System,  (3) Ill-Defined symptoms, (4) Other Medical Illness | January 1982 - December 2003  Follow up: 16 years | NA *Individuals’ diagnosed with a SUD after baseline hospitalization (Group 2) were more likely to have more medical hospitalizations and to be diagnosed with schizophrenia compared to those who were diagnosed with a SUD, including co-occurring disorders, at baseline (Group 1).  *91% of total hospitalizations over the 16-year period for Group1 were to psychiatric inpatient care, in Group 2 77% (91± 17). Group1 had significantly higher rates of psychiatric rehospitalisation than Group2 (p < .0001) | NA Individuals without SUD at baseline (Group 2) were more likely present at baseline with other medical illnesses in compare to those who had no SUD over the 16- year period (61% vs 11%)  More likely to present at baseline with other medical illnesses, less likely to be diagnosed with a mood disorder at baseline psychiatric hospitalization | No sign. differences between Group 1 (SUD at baseline) vs (Group 2 -without SUD at baseline) in medical diagnosis: Endocrine, nutritional and metabolic diseases, immunity disorders (29% vs 28%) Circulatory system (29% vs 31%)  Ill-defined symptoms (22% vs 19%) Other medical illnesses (69% vs 69%) Patients in Group 2 were significantly more likely to have a co-occurring diagnosis of schizophrenia and substance abuse than those in Group 1 |
| 6. | Li et al, 2008  [53] | To determine the rate and factors associated with seeking readmission among the clients admitted to an inpatient medical withdrawal management program and examine of the potential risk factors associated with short-term (1 month) and long-term (2 to 12 months) requests for readmission simultaneously | Yes | The most frequently self-reported primary preferred substance at admission was alcohol (55%), followed by cocaine (29%), and opiates (14%). Approximately 29% of clients were poly-drug users (e.g., using 2 or more drugs) | Drug-related diseases: HIV positive, Hepatitis C (31% clients were reported to have HCV and 8% HIV infection) | July 2003 - June 2004  Follow up: 12 months | 43% requested readmission within the 1-year. Among these clients, 46 (4%) requested readmission to VD within 1 month  *whose primary preferred substance was alcohol, and those who were poly-drug users were more likely to request readmission in long-term | Clients who reported to have hepatitis C virus (HCV) infection, were more likely to request readmission in long-term (less likely to request readmission within 1 month, but were more likely to request readmission between 2 and 12 months) | Clients who reported to have HCV, and who reported alcohol as a primary preferred substance were more likely to request readmission in short-term and long-term periods than those who did not report these two conditions |
| 7. | Irmiter et al, 2007  [34] | To examine the reinstitutionalization across a range of care settings (medical inpatient, nursing home, residential psychiatric and substance use) of patients with schizophrenia, schizoaffective disorder and bipolar disorder | Yes | Serious mental illness (SMI): schizophrenia, schizoaffective disorder, bipolar disorder | Charlson score for medical comorbidity | 1998 - 2005  Follow up: 96 months | 86% were reinstitutionalized to any VA setting. * from them 68% were reinstitutionalized to inpatient psychiatric units, 20% to inpatient non-psychiatric units, 1% to nursing homes, 6% to community residential or domiciliary/vocational care settings, 3% to psychiatric residential rehabilitation, and 2% to inpatient substance abuse/dependence units | Patients were at increased risk of reinstitutionalization if they had higher Charlson comorbidity index scores | *Mortality: specified only for the population not rehospitalised (very low) :2%  *Of individuals reinstitucionalized 73% were hospitalised for psychiatric care and 20% for non-psychiatric or medical care. *Reinstitutionalization to inpatient psychiatric care occurred sooner (mean days 326, SD = 505) than for patients reinstitutionalized to other settings (mean days 522, SD = 679; p < 0.001). Those readmitted to inpatient psychiatric care (73%) were admitted on average within 1 year, and those reinstitutionalized to other settings (27%) were admitted on average within 1.5 years |
| 8. | Clements et al, 2006  [54] | Comparing the self-report Behavior and Symptom Identification Scale (BASIS-32) and clinical-rated Global Assessment of Functioning (GAF) in their ability to predict a measure of a 1-year psychiatric hospital readmission | Yes | DSM-IV-TR: Axis I and Axis II diagnoses; number of psychiatric diagnoses (variable: 1 or more than 1) | Medical comorbidity (DSM-IV: Axis III) – variable: yes/no | 1 January 1998, 31 December 1999  Follow-up: 1 year | 33%;  23% rehospitalised due to psychiatric reasons;  8% medical hospitalisations | Medical comorbidity was not related to rehospitalisation | *Self-report was better than clinician rating in predicting psychiatric outcome (readmission)  *Readmitted patients were more likely to be unemployed, have had 1 or more previous admissions, higher admission BASIS-32 score and longer length of stay compared to the subjects who were not readmitted within 1 year |
| 9. | Morrow-Howell et al, 2006  [55] | The study addressed factors associated with six-month post-acute dispositions (continuous community stay, medical hospitalization, psychiatric rehospitalisation, nursing home placement, death) for older adults hospitalized for depression and discharged to the community | Yes | DSM-IV diagnosis – all subtypes of depression (85% - major depression; 11% - bipolar-depressive phase, 5% depression not-otherwise-specified), 16% - diagnosis of dementia | Severity of medical conditions (Chronic Illness Rating Scale-Geriatric)  Psychical Health Service Use (number of visits) | March 1997 – May 1999  Follow-up: 6 months | 23% - psychiatric rehospitalisation;  8% medical hospitalization | Medical condition was not related to rehospitalisation | *Mortality: 3%.  *Physical function (OARS) assessment at discharge was related to disposition  *Those at higher risk of psychiatric re-admission had more previous psychiatric hospitalizations and were marginally more likely to be married and have lower Brief Psychiatric Rating Scale scores at discharge |
| 10. | Thomsen & Kessing, 2005  [56] | To examine whether hospitalization with depressive disorder or bipolar disorder was a risk factor for development of hyperthyroidism | Yes | Depressive disorder: (ICD-8 diagnoses 296.09, 296.29, 296.89, 296.99 and ICD-10 diagnoses: F32.0-F33.31, F34.0-F34.9, F38.1-F39.9)  Bipolar disorder (ICD-8 codes:296.19, 296.39 and ICD-10 codes: F30.0-F31.6,F.38.0) | Diagnosis of hyperthyroidism (ICD-8 codes: 242.09-242.29 and ICD-10 codes: E03.2-E03.9)  Control group: diagnosed with osteoarthritis (ICD-8 diagnoses: 713.00–713.09, and ICD-10 diagnoses: M15.0- M19.9) | 1 April 1970 - 31 December 1993  Follow up: from the first admission in the study period | 133 570 patients discharged with an index diagnosis - 610 patients were later readmitted.  For patients with an index diagnosis of depression, the median time from index discharge to readmission with a diagnosis of hyperthyroidism was 6.58 years (range 0.02–21.38 years)  For patients with an index diagnosis of bipolar disorder, the median time to readmission with hyperthyroidism was 4.82 years (range 0.12– 19.60 years)  For patients with an index diagnosis of osteoarthritis, the median time to readmission with hyperthyroidism was 5.79 years (range 0.01– 20.09 years) | Patients with bipolar disorder were at an increased risk of subsequent hospitalization with hyperthyroidism, whereas patients with depressive disorder were not  The hazard ratio of hyperthyroidism was 1.86 (CI 95%: 1.13–3.06; p > 0.01) greater for individuals with an index diagnosis of bipolar disorder compared to individuals with an index diagnosis of depression | The total number of hospital admissions with hyperthyroidism was relatively low. Only 0.64% of patients with a discharge diagnosis of bipolar disorder and only 0.42% of patients with a discharge diagnosis of depression were later hospitalized with a main diagnosis of hyperthyroidism |
| 11. | Kessing et al, 2004  [57] | To examine whether patients hospitalised for depressive or bipolar disorders are at increased risk of getting a diagnosis of diabetes at readmission compared to patients previously admitted for osteoarthritis | Yes | Depression was defined as a main diagnosis of ICD-8 code 296.09 or 296.29 or ICD-10 code DF32.0–DF33.31  Mania/bipolar illness was defined as ICD-8 code 296.19 or 296.39 or ICD-10 code DF30.0–DF31.6 or DF38.00 | Diabetes was defined as a main diagnosis of ICD-8 code 249.00–250.09 or ICD-10 code DE10.0–DE11.9  Osteoarthritis was defined as a main diagnosis of ICD-8 code 713.xx or ICD-10 code DM15.0–DM19.9 | 1. January 1977- 31. December 1997 | The total readmission rate was calculated as the number of readmissions divided by the number of person-years at risk for readmission  Mania/bipolar disorder: 0.83 admissions/person year *depression: 0.67 admissions/person year *osteoarthritis 0.14 admissions/person year | Overall, the risk of getting readmitted with diabetes was not increased for patients who had previously been admitted with depression or mania/bipolar disorder compared to patients with osteoarthritis  There was no difference in the risks of developing Type 1 and Type 2 diabetes | *Mortality: The proportion of patients who died within the observation period was larger among patients with osteoarthritis (32.7%) than among patients with depression (28.2%) and patients with mania/bipolar disorder (23.0%)  *Patients in age groups between 45 and 80 years of age who were discharged with a diagnosis of mania/bipolar disorder had a slightly increased rate (not sign.) of getting a diagnosis of diabetes compared to patients discharged with a diagnosis of osteoarthritis whereas younger and older patients with mania/bipolar illness had a slightly decreased rate of diabetes *For women who were discharged with a diagnosis of mania/bipolar disorder at the age of 35 the probability of getting a diagnosis of diabetes was lower during the first 20 years (from 35 to 55 years of age) and higher during subsequent years. For older women discharged with mania/bipolar disorder and for patients discharged with depression the probabilities of getting a diagnosis of diabetes was not increased compared to patients with osteoarthrosis |
| 12. | Seager et al, 2000  [46] | To examine in group of persons with learning disabilities the number of readmissions and the reasons for these readmissions | Yes | ICD-10 psychiatric diagnosis  * The diagnoses (other than of learning disability) for the study population other than of learning disability (No of patients): Autism -7 Dementia- 6 Schizophrenia- 2 Schizophrenic/delusional episode (DC-LD diagnosis)- 2 Schizoaffective disorder (DC-LD diagnosis)- 1 Bipolar disorder- 2 Recurrent depressive disorder- 5 Simple phobia-1 *Where it was not possible to make a psychiatric diagnosis using ICD-10 criteria, owing to the level of learning disability of a patient, the newly developed classification system The Diagnostic Criteria for Psychiatric Disorders for Use with Adults with Learning Disabilities/Mental Retardation (DC-LD) was used instead. * The level of learning disability ranged from borderline to profound, with 73% being severely or profoundly learning disabled | Down’s syndrome, developed dementia, stroke (cerebrovascular accident resulting in some weakness), a fall associated with a degree of paralysis and very poorly controlled epilepsy | 1981 - 1999  Follow up: 18 years (the majority of discharges occurred over the last 5 years, 6 patients were discharged in 1980s and 11 patients in the early 1990s | 8.4% (N=13) | Physical deterioration figured highly as a cause of readmission to the learning disability hospitals  Of those readmitted because of a deterioration in their physical health (N=6), 2 people had Down’s syndrome and had developed dementia, 1 had a probable stroke (cerebrovascular accident resulting in some weakness), 1 had a fall associated with a degree of paralysis and 2 had very poorly controlled epilepsy | *Readmission to hospital from community facilities occurred most commonly as a result of physical aggression. 9 of readmissions-of those whose behaviour had been sufficiently disturbed and, usually, sufficiently dangerous to render continuing residence in the community an impossibility with their existing level of staffing. The other group (six of the readmissions) comprised individuals who had been readmitted because of deterioration in their physical health  *The results showed a non-significant trend (p < 0.5, chi-squared, 1 d.f.) towards higher rates of readmission among individuals with lesser (borderline to moderate) degrees of learning disability |
| 13. | Brennan et al, 2000  [59] | To examine the characteristics and 4 -year readmission of elderly inpatients with substances use disorders, determined whether their readmission are elevated relative to case controls and analysed gender differences in characteristics and predictor of readmissions | Yes | Substance abuse, dependence, psychosis diagnosis: (ICD-9 codes 291, 292, 303, 304, 305.0, 305.2-305.9) or an ICD-9 surrogate medical diagnosis indicative of alcohol abuse or dependence (following Adams et al., 1993: codes 265.2, 357.5, 425.5, 535.3, 571.0-571.39, 790.3, E860.0-E860.1).  88% patients had a substance abuse, dependence or psychosis diagnosis, 12% had only a surrogate alcohol-related diagnosis | Number of medical diagnosis, Surrogate medical diagnosis indicative of alcohol abuse or dependence, Accident-related diagnosis: accidentional poisonings, adverse drug reactions, accidental falls.  Almost 9% of patients had an accident-related diagnosis. 75% of these accidents were accidentional poisonings and adverse drug reactions and additional 20% were accidental falls | 1991-1994  Follow up: 48 months | Cumulative 4 -year readmission rate was 73%. (M: 72.4%, F: 74.2%). Of readmitted patients 68% had multiple readmissions  45 % were rehospitalized with a mental health diagnosis (M: 44.3%, F: 45.6%)  54% of patients were rehospitalized with a medical diagnosis (M: 52.9%, F: 54.8%) | Fewer medical diagnoses increased risk of mental health readmissions over the next 4 years (both gender) | *Mortality: The HCFA Health Insurance Skeleton Write off file (Kestenbaum, 1992) was used to identify patients who were still alive 4 years after discharge.  *From 22 768, alive after 4 years 12 417  *Index episode characteristics in average number of medical disorders: 3.5 (M: 3.5, F: 3.4);  * Accidents were implicated in a higher proportion of women's index episodes. Accidents-all types: 8.9% (M: 6.8%, F: 13.3%); Accidental poisoning and adverse drug reaction: 6.7% (M: 4.1%, F: 10.9%); Falls: 1.8% (M: 1.6%, F: 2.2%). *Among both gender earlier hospitalizations involving substance use disorders, dual diagnosis and more severe psychiatric disorders were associated with higher risk of readmission |
| 14. | Labbate & Doyle, 1997  [60] | To determine differences between patients admitted multiple times compared with once for major depressive disorder (MDD) | Yes | Major depression DSM III-R | Medical illness (not specified)  With medical illness: recidivist- 57%; single admission -34% | 1991-1995  Follow up: 48 months | 21% of admitted patients with MDD were admitted at least twice  8% of patients were admitted three or more times over a 4-year period | Medical illness statistically more common among recidivists than single admission patients; recidivist: 57% with medical illness | In this sample was found that for patients with MDD, recurrent episodes of depression, older age, and use of ECT also predicted recidivism |
| 15. | Phibbs et al, 1997  [61] | The study focused on developing of a case mix model for inpatient substance abuse treatment to assess the effect of case mix on readmission across Veterans Affairs Medical Centers (VAMCs) | Yes | ICD-9-CM diagnosis codes were used to identify patients receiving treatment for substance abuse | ICD-9-CM codes: heart, arthritis, anaemia, diabetes, back, bronchitis, other lung, cancer | Fiscal years 1987 - 1992  Follow up: 6 months (within 180 days of discharge) | Overall, 26.4% of the 1990 substance abuse discharges from VA were readmitted within 180 days | Chronic lung conditions increase the probability of readmission by an additional 1.7% | *A patient who has no medical or psychiatric comorbidities, no other substance problems, and no previous treatment episodes in the past year-probability of readmission within 180 days is about 18%. The probability of readmission increases to 30% if the patient has had a treatment episode within the last year and to 62% if the patient has had three or more treatment episodes in the last year.  *A psychiatric comorbidity of schizophrenia, manic, bipolar, or post-traumatic stress disorder increases the probability of readmission by an additional 10%.  *An opiate or cocaine use disorders increases the probability of readmission by 2%. |
| 16. | Kent & Yellowlees, 1994  [62] | To identify factors that commonly contributed to the decision to hospitalize patients who made heavy use of mental health services | Yes | Schizophrenia, schizoaffective disorder, bipolar disorder, personality disorders (as comorbidity and as primary diagnosis)  42 of subjects (84 %) were diagnosed as having a major functional psychiatric disorder on axis I of DSM-iii-R, and 11 had more than one axis I disorder. Schizophrenia was the most frequent axis I illness, affecting 16 patients, followed by schizoaffective disorder (12) and bipolar disorder (10) patients. Personality disorder was present in 21 cases and was the primary diagnosis in five cases | Physical health problems (not specified) | January 1989 - December 1991  Follow up: 36 months | 50 patients accounted for a total of 442 admissions over the three-year study period (M: 8.8; SD: ±6.8 admissions)  The number of admissions per patient ranged from 3 to 40 | Physical health problems contribute to the decision to readmit in 16.5% of admissions | When the 15 factors were combined into four major categories, social factors were found to contribute to 38.9% of admissions, followed by factors related to psychiatric and physical illness (31.1%), dangerousness to self or others (20.3%), and substance abuse (9.7%) |
| 17. | Colenda et al, 1991  [63] | The study examined in three groups of depressed patients: late-onset geriatric patients, early-onset geriatric patients, and young adult patients:  (1) differences in hospital utilization patterns and admission clinical symptoms (2) differences in readmission rates  (3) prediction of readmission according to specific clinical symptoms on admission | Yes | DSM-III-R criteria for unipolar major depression based on the DSM-Ill-R check list of recorded symptoms and signs  * 3 groups of patients: 1. Late-onset geriatric depression (LOGD): The first episode of depression began after age 60 years 2. Early-onset geriatric depression (EOGD): The first episode of depression began in midlife, but at the time of the index MCV admission the patient was over age 55 years 3. Young adult depression (YAD): The first episode of depression began between ages 20 and 40 years and at the time of the index MCV admission the patient was less than age 45 years | Number of medical diagnoses and somatic complaints  Somatization was defined as excessive concern with body or functional capacity not deemed to be associated with a medical illness or, if associated with a medical illness, the somatic complaints exceeded expected limitations | Follow up: 24 months | The 24-month readmission rates for LOGD patients was 47.4%, and differed significantly from both, EOGD patients (23.1%) and YAD patients (16.7%) | Patients with somatic complaints have an increased relative risk for readmission within 24 months  For LOGD and EOGD patients, the risk of readmission was greatest for those patients who had somatic symptoms at the time of admission (M: 1.7 and 5, respectively) | * The number of medical diagnoses (M, SD) differed significantly across the three groups i.e., geriatric patients had more medical problems than young adults, but both geriatric groups had similar levels of medical comorbidity: LOGD : 2.8 (1.9); EOGD: 2.2 (1.8); YAD: 0.8 (1.2) *Hospital utilization patterns for the YAD were different from the geriatric patients, but in the expected direction, i.e., less medical comorbidity and shorter lengths of stay |
| 18. | Walley et al, 2012  [64] | To determine whether having a substance use disorder diagnosis was associated with subsequent acute care hospital utilization. Rate and risk of acute care hospital utilization (emergency department visit or hospitalization) were evaluated within 30 days of discharge | No *Medical problems recorded only at admission not at readmission analysis | ICD-9 Diagnostic codes included 303 (alcohol intoxication and, 305.0 (alcohol abuse), 291 (alcohol-induced mental disorders), 304 (drug dependence), 305.2 to 305.7, 305.9 (drug G4abuse), and 292 (drug-induced mental disorders)  For exploratory analyses, the substance use disorders variable was split into 3 categories: alcohol diagnosis only, drug diagnosis only, and concurrent alcohol and drug diagnoses | Charlson Comorbidity Index score | January 2006 - October 2007  Follow up: 1 month | Patients with substance use diagnoses, specifically drug use diagnoses, have higher acute care hospital reutilization (33% vs 22% at 30 days, P < 0.05) | NA | Subjects with a substance use disorder diagnosis had higher rates of recurrent acute care hospital utilization than patients without substance use disorder diagnoses (No. of visits/patient/30 days: 0.63 vs 0.32 events per subject at 30 days, P < 0.01) |
| 19. | Mai et al, 2011  [65] | To compare mental health clients (MHCs) with non-MHCs on potentially preventable hospitalisations (PPHs) as an indicator of received quality of primary care | No *Medical problems recorded only at admission not at readmission analysis | Alcohol/drug disorders, Schizophrenia, Affective psychoses, Other psychoses, Neurotic disorders, Personality disorders, Adjustment disorders, Depressive disorders, Other mental disorders | Charlson physical comorbidity score  Four PPH medical conditions:  1) Influenza and pneumonia, other vaccine-preventable  2) Chronic (Asthma, Congestive heart failure, Diabetes complications, COPD, Angina, Iron deficiency anaemia, Hypertension, nutritional deficiencies, Rheumatic heart disease  3) Acute (Dehydration and gastroenteritis, Pyelonephritis, Perforated/bleeding ulcer, Cellulitis, Pelvic inflammatory disease, Ear, nose and throat infections, Dental conditions, Appendicitis with generalised peritonitis, Convulsions and epilepsy, Gangrene; 4) Adverse drug events | 1990 to 2006  Follow up: 16.5 years (from 1. Jan 1990 to 30. June 2006) | NA | NA  PPHs accounted for more than 10% of all hospital admissions/discharges in MHCs, with diabetes and its complications, adverse drug events (ADEs), chronic obstructive pulmonary disease (COPD), convulsions and epilepsy, and congestive heart failure being the most common causes | *Relative to non-MHCs, MHCs have more physical comorbidities (Charlson score: 1.04 (2.24) vs 1.28 (2.34), p < 0.001)  *On average, MHCs were about twice as likely as non-MHCs to experience a PPH, with the largest differences occurring in PPHs for convulsions and epilepsy, nutritional deficiencies, ADEs, COPD and asthma  *Rate of PPHs were higher in those with relatively more debilitating mental disorders such as alcohol and drug disorders, affective psychoses, other psychoses and schizophrenia  *Potentially preventable hospitalisations, by selective categories of mental disorders: 1) Alcohol and drug disorders: Nutritional deficiencies, Convulsions and epilepsy, Gangrene, COPD, Other vaccine-preventable conditions, Perforated/bleeding ulcer  2) Affective disorders: Nutritional deficiencies, Convulsions and epilepsy, Adverse drug events, Asthma, COPD;  3) Other psychoses: Convulsions and epilepsy, Pyelonephritis, Adverse drug events, Other vaccine-preventable conditions, Gangrene  4) Schizophrenia: Nutritional deficiencies, Adverse drug events, Convulsions and epilepsy, Congestive heart failure, Influenza and pneumonia |
| 20. | Hassan & Lage, 2009  [65] | To examine the relationship between non-adherence to antipsychotic medication after hospital discharge and risk of rehospitalisation in patients who were previously hospitalized for treatment of bipolar disorder | No *Medical problems recorded only at admission not at readmission analysis | Patients with bipolar disorder- International Classification of Diseases, 9th Revision, Clinical Modification (ICD-9-CM) diagnosis of 296.4x–296.8  Accompanying mental health diagnosis (ICD- 9-CM code 290.xx–319.xx) panic disorder, obsessive compulsive disorder, generalized anxiety disorder, attention-deficit/hyperactivity disorder, depression, substance abuse | Charlson comorbidity score    Obesity, cardiovascular disease, diabetes, hypertension, high cholesterol | 1. January 2000 - 31. December 2006  Follow up: 12 months | NA  Being diagnosed as bipolar type depressed, and having a pre-index period comorbid diagnosis of depression were all significantly associated with both a higher risk of rehospitalisation and a higher risk of a mental-health-related rehospitalisation | NA  Medical condition recoded at admission:  Hypertension 19.3% Diabetes mellitus 6.5% High cholesterol 5.8% Obesity 5.4% Cardiovascular disease 4.3%  Charlson comorbidity score recorded during pre-index period (0.48 ± 0.99) | Among patients who were previously hospitalized for treatment of bipolar disorder, those who were adherent to their antipsychotic medication at least 75% of the time had lower risks of all-cause rehospitalisation and mental-health-related rehospitalisation |
| 21. | Callaghan & Cunningham, 2002  [67] | The main interests of the study were: to examine gender differences among those admitted to an inpatient detoxification; to construct a multiple logistic regression model for men and women predictive of detoxification dropout; and to build a statistical model predicting re-admission to the detoxification treatment program | No *Medical problems recorded only at admission not at readmission analysis | Primary drug of choice reported at admission (M/F): Alcohol- 62.9%/53.5% Cocaine- 20.6%/22.8% Opiates- 9.0%/14.7% Benzodiazepines- 1.3%/4.5% Other- 6.2%/4.5% | Medical problems at admission (M/F) Hepatitis B- 6.5%/4.4%  Hepatitis C- 20.7%/25.9%  HIV(+) - 1.1% /2.9%  Cardiac problems/angina - 9.6%/5.9%  Cirrhosis -6.7%/6.7%  Hypertension -19.0%/11.9%  Asthmatic -11.7%/22.4%  Gastritis - 18.3%/15.6%  Diabetic - 6.2%/4.7%  Pregnant - 0.0%/2.7% | January 1999 -January 2002  Follow up: 36 months | NA | NA | * At admission: females were administered prescription medication (i.e., anti-depressants, antibiotics) and medical evaluation test (e.g., X-rays, blood tests) at a significantly higher rate than males  *Significant predictors of detoxification re-admission were: residential instability, alcohol as a primary drug of choice, single marital status, unemployment, multiple drug use, an older age (>37 years), Aboriginal ethnicity and treatment incompletion at Time 1. Gender was not a significant factor) |
| 22. | Mercer et al, 1999  [68] | To identify a combination of variables that could predict rehospitalisation of older psychiatric inpatients | No *Medical problems recorded only at admission not at readmission analysis | All Psychiatric diagnoses were coded on all five axes of the Diagnostic and Statistical Manual of Mental Disorders (4th ed., or DSM-IV, American Psychiatric Association, 1994) multiaxial system | Medical burden as number of medical diagnoses at admission/discharge included coronary artery disease, hypertension,  and diabetes  The control group had a slightly (non sign.) higher number of medical illnesses than did the rehospitalisation group (4.4 vs 3.9). Groups were not statistically different on medical burden (i.e., number of medical illnesses) | October 1993- March 1996  Follow up: 18 months | NA | NA | *Psychiatric patients were found to have approximately four times more psychiatric hospitalizations than medical hospitalizations despite the existence of multiple medical illnesses in this population *More hospitalized patients were diagnosed with schizophrenia and mood disorders  *Patients' psychiatric diagnosis (mood or schizophrenic disorder), poor general psychiatric functioning, depressive and agitated behaviour at discharge, little or no supervision in living arrangements following discharge, limited social support, change in the social support system preceding hospitalization, and maladaptive family functioning could significantly predict geropsychiatric rehospitalisation. The strongest independent predictor was maladaptive family functioning |
| 23. | Walker et al, 1995  [69] | To examine the prevalence of substance use, psychiatric, and medical disorders in mark discharged from Veterans Affairs hospitals | No *Medical problems recorded only at admission not at readmission analysis | ICD-9 codes relevant to substance use disorders for alcohol dependence (code 303), alcohol abuse (code 305.0), alcoholic psychosis (code 291), drug dependence (code 304), drug abuse (codes 305.2-305.9), and drug psychosis (code 292) | Medical diagnoses at admission: skin and subcutaneous tissue diseases, infectious parasitic diseases, digestive diseases | October 1990 - March 1991  Follow up: from April 1991- September 1991 (for at least 183 days in fiscal year) | 44 % of women with substance use disorders who were discharged during the first six months of fiscal year 1991 were rehospitalised within that year | NA | *The mean± SD number of inpatient discharges was 2±1.7 for women with substance use disorders, 1.6±1.2 discharges for women without such disorders. *Readmitted to an addictions, psychiatric, or medical-surgical service in a VA hospital by the end of the fiscal year: 34.7% received the substance-related diagnosis from an addictions treatment unit, 53.4% received it from a psychiatric unit, and 41.8% received it from a medical or surgical unit  *Skin and subcutaneous tissue diseases, infectious and parasitic diseases, and digestive diseases were more prevalent (per 100 patients) among women with substance use disorders than among women in the same age group (34.4±4.6) who did not have substance use disorders discharged from VA hospitals: Blood and blood-forming organs 6.2/6.2 ; Circulatory system 8.1 /13.6; Musculoskeletal system and connective tissue 9.1/18.9 ; Skin and subcutaneous tissue 8.0/4.9 ; Genitourinary system 10.9/21.0; Endocrine, nutritional, metabolic, and immune systems 11.3/14.4 ; Infectious and parasitic disease 13.2/ 8.5 ; Nervous system and sense organs 9.7/ 11.7 |
